# PSD-95-nNOS Coupling Regulates Contextual Fear Extinction in the Dorsal CA3

**DOI:** 10.1038/s41598-018-30899-4

**Published:** 2018-08-24

**Authors:** Cheng-Yun Cai, Chen Chen, Ying Zhou, Zhou Han, Cheng Qin, Bo Cao, Yan Tao, Xin-Lan Bian, Yu-Hui Lin, Lei Chang, Hai-Yin Wu, Chun-Xia Luo, Dong-Ya Zhu

**Affiliations:** 1 Institution of Stem Cells and Neuroregeneration, Nanjing Medical University, Nanjing, 211166 People’s Republic of China; 2 Departments of Pharmacology, School of Pharmacy, Nanjing Medical University, Nanjing, 211166 People’s Republic of China; 3 The key laboratory of human functional genomics of Jiangsu Province, Nanjing, 211166 People’s Republic of China

## Abstract

Fear extinction depends on N-methyl-D-aspartate glutamate receptors (NMDARs) and brain-derived neurotrophic factor (BDNF) activation in the limbic system. However, postsynaptic density-95 (PSD-95) and neuronal nitric oxide synthase (nNOS) coupling, the downstream signaling of NMDARs activation, obstructs the BDNF signaling transduction. Thus, we wondered distinct roles of NMDAR activation and PSD-95-nNOS coupling on fear extinction. To explore the mechanisms, we detected protein-protein interaction using coimmunoprecipitation and measured protein expression by western blot. Contextual fear extinction induced a shift from PSD-95-nNOS to PSD-95-TrkB association in the dorsal hippocampus and c-Fos expression in the dorsal CA3. Disrupting PSD-95-nNOS coupling in the dorsal CA3 up-regulated phosphorylation of extracellular signal-regulates kinase (ERK) and BDNF, enhanced the association of BDNF-TrkB signaling with PSD-95, and promoted contextual fear extinction. Conversely, blocking NMDARs in the dorsal CA3 down-regulated BDNF expression and hindered contextual fear extinction. NMDARs activation and PSD-95-nNOS coupling play different roles in modulating contextual fear extinction in the hippocampus. Because inhibitors of PSD-95-nNOS interaction produce antidepressant and anxiolytic effect without NMDAR-induced side effects, PSD-95-nNOS could be a valuable target for PTSD treatment.

## Introduction

Learning about potential dangers in the environment is critical for adaptive function, but the fear learning for emotional disorders, post-traumatic stress disorders (PTSD) in particular, can be maladaptive, resulting in excessive fear and anxiety^1^. PTSD is extraordinarily robust and difficult to treat, because of enhanced fear learning, impaired extinction or inability to modulate fear expression using contextual information^2^. Extinction, the learned inhibition of retrieval, is widely used in the treatment of PTSD, often under the term “exposure therapy”^3^. The extinction learning involves new learning of an inhibitory signal that competes with the previously learned fear memory^4^. Contexts, a set of circumstances around an event, are essential for abstracting situationally informed meaning from the world. Contextual processing deficits are at the core of PTSD pathophysiology^1,2^. Hippocampus has a crucial role in tasks involving learning and remembering contexts^1^. Therefore, understanding molecular pathways mediating contextual extinction learning in the hippocampus is particularly important to treat the disorder.

Convergent evidence from animal and human studies suggests that extinction of recently and remotely acquired fear depends on N-methyl-D-aspartate glutamate receptor (NMDAR) activation in the hippocampus, basolateral amygdala and ventromedial prefrontal cortex^5–7^. Each NMDAR is a calcium-permeable tetrameric ionotropic receptor complex consisting of two obligatory GluN1 subunits and two GluN2 (A-D) or GluN3 (A, B) subunits^8^. In the adult forebrain regions, GluN2A and GluN2B subunits are the main subunits available in excitatory synapses for receptor complex formation^9^. GluN2B-containing receptor has a preferential role in the induction of synaptic plasticity critical for the extinction of fear memories^10^. The carboxyl terminus of each subunit binds important intracellular signaling complexes, allowing for their efficient and selective activation by calcium influx through the opening of NMDAR channels^11^. One of the well-characterized intracellular signaling complexes of GluN2B is the PSD-95-nNOS complex, in which, the protein postsynaptic density-95 (PSD-95) is a scaffolding protein that links GluN2B carboxyl terminus to neuronal nitric oxide synthase (nNOS) at excitatory synapses^12^. Activation of nNOS depends on its association with PSD-95 and on NMDAR-mediated calcium influx^13^. We recently found that the PSD-95-nNOS signaling complex impairs neuroplasticity, including neurogenesis, spine growth and dendrite development^14^, which is clearly different from the role of NMDAR activation. Given that neuroplasticity is crucial for memory extinction^15^, we hypothesized that NMDAR activation and PSD-95-nNOS coupling may play different role in the modulation of contextual fear extinction in the hippocampus.

Brain-derived neurotrophic factor (BDNF), a member of the neurotrophin family identified as a critical factor that mediates synaptic plasticity associated with learning and memory, specifically in fear learning and extinction^16^. The functions of BDNF are mediated by the receptor tyrosine kinase TrkB, which is present in the fraction of postsynaptic density in the adult rat brain^17^. Upon NMDARs activation, PSD-95 not only interacts with nNOS to form PSD-95-nNOS complex^13^, but also with TrkB to form PSD-95-TrkB complex^17,18^ at excitatory synapses. Based on previous reports, we speculated that nNOS and TrkB may compete with each other to form complexes with PSD-95, thus playing an important role in fear extinction.

Extracellular regulated protein kinase (ERK) regulates hippocampal histone following contextual fear conditioning^19^. NO produced from nNOS in the presence of L-arginine is a potent inhibitor of Ca^2+^-mediated ERK activation^20^. Therefore, ERK activation may contribute to the role of PSD-95-nNOS in regulating BDNF expression.

In general, we hypothesized that disassociating PSD-95-nNOS coupling in the hippocampus may up-regulate BDNF expression via inhibiting ERK activation, enhanced the association of BDNF-TrkB signaling with PSD-95, and promoted contextual fear extinction. Conversely, blocking NMDARs down-regulated BDNF expression and hindered contextual fear extinction. NMDARs activation and PSD-95-nNOS coupling play distinct roles in modulating contextual fear extinction.

## Results

### Contextual Fear Extinction Induces a Shift from PSD-95-nNOS to PSD-95-TrkB Coupling in the Hippocampus

**Figure 1:**
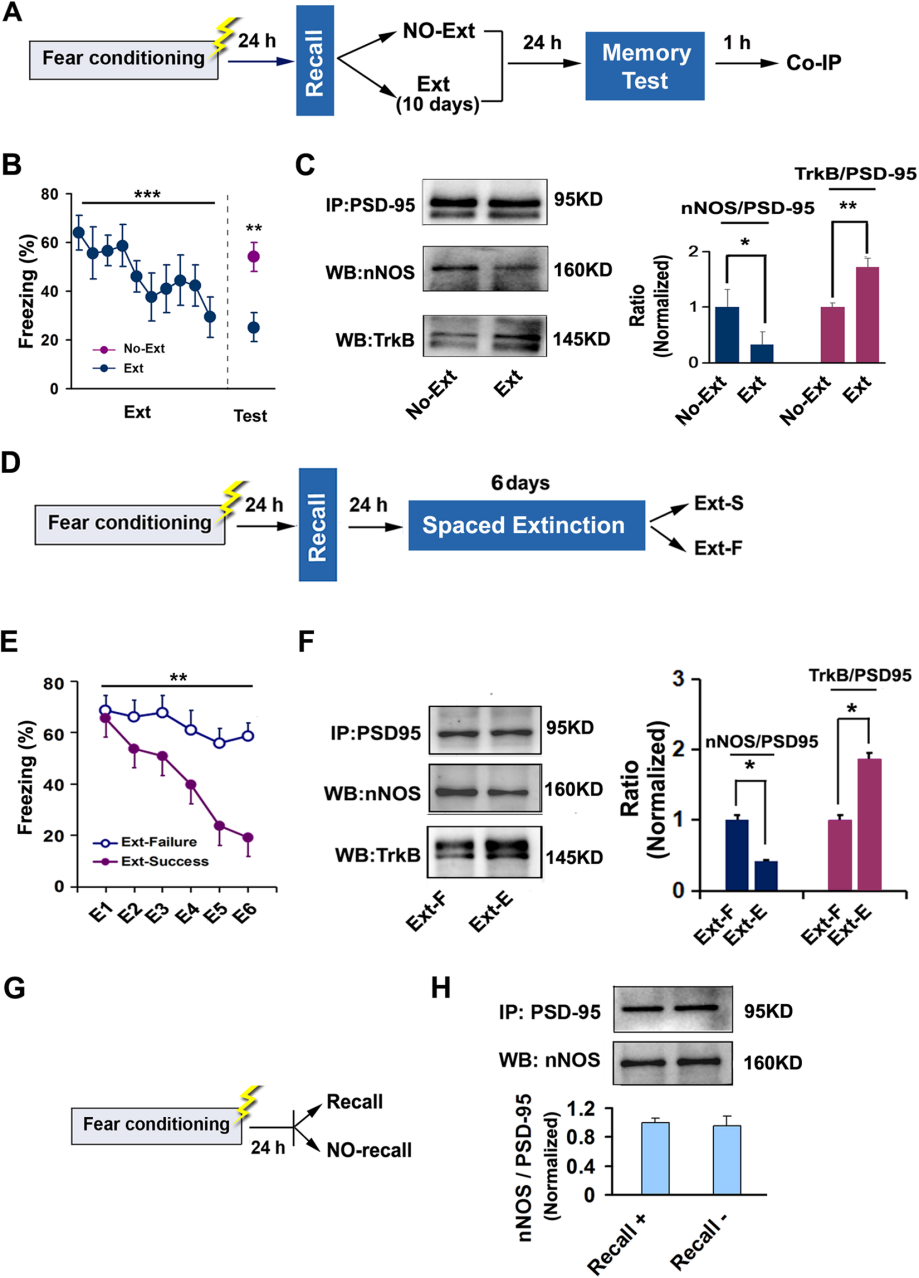
Contextual fear extinction induces a shift from PSD-95-nNOS to PSD-95-TrkB coupling in the dorsal hippocampus. (**A**) Design of the experiments for (B–D). (**B**) Freezing behavior measured during extinction trial and retrieval of extinction memory (n = 6). (**C**) PSD-95-nNOS and PSD-95-TrkB complex levels in the dorsal hippocampus after contextual fear extinction. (PSD-95-nNOS: n = 5; PSD-95-TrkB: n = 5). (**D**) Design of the experiments for (E and F). (**E**) Freezing behavior measured during extinction trial from the mice with successful extinction and failured extinction (n = 5–6). (**F**) PSD-95-nNOS and PSD-95-TrkB complex level in the dorsal hippocampus after contextual fear extinction (PSD-95-nNOS: n = 5–6; PSD-95-TrkB: n = 5–6). (**G**) Design of the experiments for (H). (**H**) PSD-95-nNOS complex level in the dorsal hippocampus after recall (n = 3). Ext: extinction. Ext-S: extinction success. Ext-F: extinction failure.

To test whether contextual fear extinction affects the interactions of PSD-95 with nNOS and TrkB in the hippocampus, we trained mice in the contextual fear-conditioning procedure as previously described^21^. All animals acquired contextual conditioned fear to the same extent as indicated by high freezing levels in response to the context at the end of the conditioning session and in the fear recall session performed 24 h later (data not shown). Next day after recall, the mice were randomly divided into extinction and no-extinction groups. Mice in extinction group were subjected to a daily extinction trial for 10 consecutive days, while no-extinction animals stayed in the home cages. Each extinction trial consisted of a 3 min re-exposure to the conditioned context without presenting the foot shock again. Extinction memory test was performed 24 h after the end of extinction trial, and 1 h later, protein-protein interaction in the dorsal hippocampus was detected (Fig. 1A). Extinction trial significantly reduced freezing level (extinction, F(1,10) = 72.41, p < 0.001; extinction memory test, F(1,10) = 11.92, *p* = 0.006) (Fig. 1B), substantially decreased PSD-95-nNOS complex level (F(1,8) = 7.99, *p* = 0.018) and increased PSD-95-TrkB level (F(1,8) = 23.00, *p* = 0.001) (Fig. 1C) in the dorsal hippocampus.

Using the procedure above, we subjected mice to a daily extinction trial for 6 consecutive days (Fig. 1D), and detected PSD-95-nNOS and PSD-95-TrkB complex level in the dorsal hippocampus from the mice of extinction-success and extinction-failure (F(5,10) = 23.165, *p* = 0.001) (Fig. 1E). The mice with successful extinction displayed significantly reduced PSD-95-nNOS complex level (F(1,8) = 7.43, *p* = 0.026) and increased PSD-95-TrkB complex level (F(1,8) = 6.26, *p* = 0.036), compared to the mice with failed extinction (Fig. 1F). However, retrieval of contextual fear did not affect the interaction of PSD-95 with nNOS, because the mice performed recall and no recall had similar PSD-95-nNOS level (F(1,4) = 0.01, *p* = 0.910) (Fig. 1H). These data suggest that contextual fear extinction leads to a shift from PSD-95-nNOS to PSD-95‒TrkB coupling in the hippocampus.

### PSD-95-nNOS Coupling in the CA3 Regulates Contextual Fear Extinction

**Figure 2:**
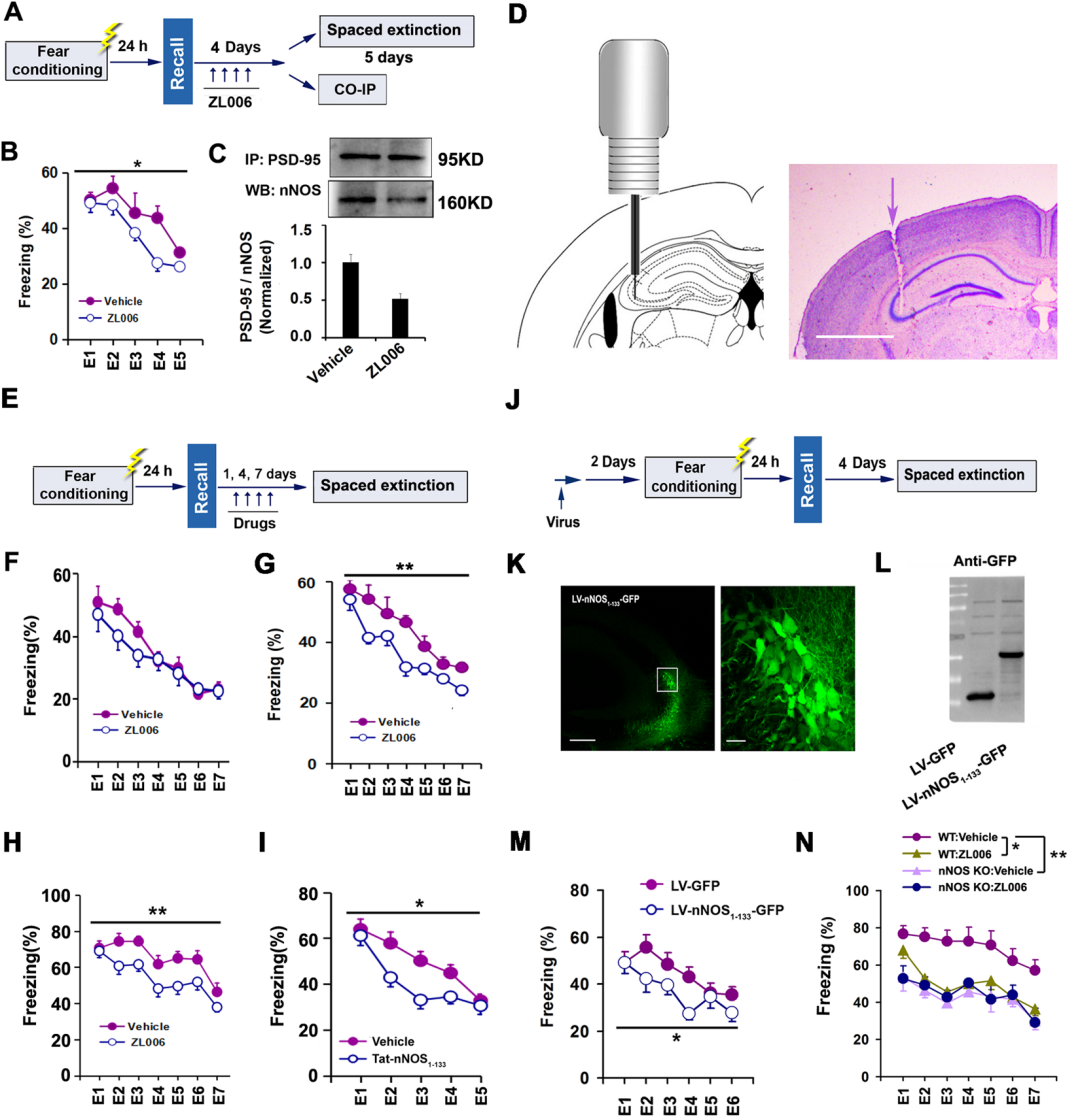
PSD-95-nNOS coupling in the CA3 regulates contextual fear extinction. (**A**) Design of the experiments for (B,C). (**B**) Effect of systemic ZL006 on fear extinction (n = 9). (**C**) PSD-95-nNOS complex level in the dorsal hippocampus (n = 5). (**D**) The representative images of micro-injection site by cresyl violet staining after every behavioural test (scale bar, 1000 μm). (**E**) Design of the experiments for (F–I). (**F**) Effect of intra-CA3 ZL006 on fear extinction (n = 8–10). (**F**) Effect of intra-CA3 ZL006 for 1 day on fear extinction (n = 13). (**G**) Effect of intra-CA3 ZL006 for 4 days on fear extinction (n = 8–10). (**H**) Effect of intra-CA3 ZL006 for 7 days on fear extinction (n = 13). (**I**) Effect of intra-CA3 Tat-nNOS_1–133_ on fear extinction (n = 10). (**J**) Design of the experiments for (K–M). (**K**) A representative fluorescence image showing the LV-nNOS_1–133-_GFP-infected CA3 (left, scale bar, 200 μm) and a high-magnification image from a selected area in the leftward image (right, scale bar, 20 μm). (**L**) Immunoblots showing nNOS_1–133_ and GFP expression in the LV-nNOS_1–133-_GFP- or LV-GFP-infected CA3. (**M**) Effect of LV-nNOS1–133-GFP in the CA3 on fear extinction (n = 12). (**N**) Effect of intra-CA3 ZL006 (using the procedure in (E)) on fear extinction in nNOS KO and WT mice (n = 11).

To test whether PSD-95-nNOS coupling regulates contextual fear extinction, we subjected mice to contextual fear conditioning as above. Two hours after fear recall, mice were treated with ZL006 (20 mg/kg/d, i.p.), a small molecular compound blocking PSD-95-nNOS binding^13^, or vehicle for 4 consecutive days. Twenty-four hours later, the mice were subjected to daily extinction for 5 consecutive days in the conditioning context (Fig. 2A). Mice treated by systemic ZL006 displayed significantly decreased levels of freezing (main group effect: F(1,16) = 4.96, *p* = 0.019), compared to vehicle, suggesting increased contextual fear extinction (Fig. 2B). Moreover, we measured PSD-95-nNOS complex level in the dorsal hippocampus 1 h after the last ZL006 injection and found that the drug significantly reduced amount of PSD-95-nNOS complex (F(1,8) = 9.84, *p* = 0.014) (Fig. 2C).

Neuronal activity causes the rapid expression of immediate early genes, such as c-Fos^22^. Accordingly, we measured c-Fos expression in the dorsal hippocampus 90 min after contextual fear recall and extinction. The number of c-Fos positive cells was increased in the CA3 and CA1, but not in the DG region among homcage, fear recall and fear extinction groups. Moreover, c-Fos induction was higher in the CA3 than in the CA1 region with extinction exposure (% increase of c-Fos-positive cells = (number in extinction group - number in homecage group)/number in homecage group × 100%, CA3 = 304.4%, CA1 = 209%) and CA1 receives the projection of CA3, suggesting dorsal CA3 may be more important in the contextual fear extinction (Supplemental Fig. S1). Furthermore, the rapid encoding of contextual memory requires the CA3 region of the hippocampus^23^. Thus, we focused on the role of PSD-95-nNOS coupling in the dorsal CA3.

Accordingly, we implanted microcannula into the dorsal CA3 in mice (Fig. 2D), and 7 days later, the mice were subjected to contextual fear conditioning. Two hours after fear recall, we infused ZL006 or vehicle through the microcannula into the CA3 of conscious mice for 1, 4 and 7 days after fear recall (Fig. 2E). From next day, the mice were subjected to a spaced extinction. Contextual fear extinction was not accelerated in mice treated with ZL006 for 1 day (main group effect: F(6,26) = 4.96, *p* = 0.376) (Fig. 2F), but significantly facilitated in mice treated with ZL006 for 4 and 7 days (Fig. 2G: main group effect: F(1,16) = 11.171, *p* = 0.004; Fig. 2H: main group effect: F(1,24) = 20.177, p < 0.001) (Fig. 2G,H). Based on the results of preliminary experiments, we employ the 4 days treatment scheme in the following experiments to investigate the effects of PSD-95-nNOS coupling on contextual fear extinction. Furthermore, the dosage scheme was based on LC-MS/MS analysis after 24 hours of intra-hippocampus infusions. The result showed that 5.935 μg/g ZL006 was detected in the dorsal hippocampus tissues after 24 hours injection (Supplemental Fig. S2). According to previous reports^13^, 0.5 μg/g ZL006 in brain tissue was sufficient to disassociate PSD-95-nNOS coupling. So the residual dosage of ZL006 after 24 hours was able to uncouple PSD-95-nNOS complex. Using the same contextual fear conditioning procedure, we infused Tat-nNOS_1–133_, a peptide blocking PSD-95-nNOS binding^13^, or vehicle through the microcannula into the CA3 after fear recall. From next day, the mice were subjected to a spaced extinction (Fig. 2E). Similarly, Intra-CA3 microinjection of Tat-nNOS_1–133_ significantly promoted contextual fear extinction too (main group effect: F(1,25) = 5.268, *p* = 0.030) (Fig. 2I). Additionally, ZL006 and Tat-nNOS_1–133_ had no effect on the spontaneous activity of mice in the open-field test (Supplemental Fig. S3A–C). To further address the role of PSD-95-nNOS coupling in the CA3, we generated a lentiviral vector that selectively expresses nNOS-N_1–133_, a region crucial for PSD-95-nNOS interaction^13^, and named it LV-nNOS_1–133_-GFP. LV-nNOS_1–133_-GFP or its control LV-GFP was microinjected into the dorsal CA3 of mice through microcannula, and two days later, the mice were subjected to contextual fear conditioning as above. Four days after fear recall, the mice received a spaced extinction (Fig. 2J). The recombinant virus effectively infected the CA3 (Fig. 2K), produced considerable nNOS-N1-133 peptide (Fig. 2L). As ZL006 and Tat-nNOS_1–133_ did, LV-nNOS_1–133_-GFP significantly promoted contextual fear extinction (main group effect: F(5,22) = 4.444, *p* = 0.047) (Fig. 2M).

To determine whether effect of drug on contextual fear extinction depends on the dissociation of PSD-95-nNOS coupling, we subjected nNOS gene knock-out (nNOS KO) or WT mice to contextual fear conditioning. Two hours after fear recall, we infused ZL006 or vehicle through implanted microcannula into the dorsal CA3 for 4 consecutive days. From next day, the mice were subjected to a spaced extinction. Although ZL006 significantly facilitated contextual fear extinction in WT mice, it had no effect on nNOS KO mice (main group effect: F(5,22) = 7.004, *p* = 0.001; *post hoc* comparisons, WT Vehicle *vs* WT ZL006, *p* = 0.022; WT Vehicle *vs* nNOS KO Vehicle, *p* = 0.001; nNOS KO Vehicle *vs* nNOS KO ZL006, *p* = 0.997) (Fig. 2N), suggesting requirement of PSD-95-nNOS coupling for behavioral effect of ZL006.

Next, we used same contextual fear conditioning procedure as the study in the CA3 and investigated the role of PSD-95-nNOS coupling in the CA1 and DG of dorsal hippocampus. Two hours after fear recall, we daily infused Tat-nNOS_1–133_ into the dorsal CA1 or DG through the microcannula for 4 consecutive days (Supplemental Fig. S4A). Surprisingly, Tat-nNOS_1–133_ did not affect fear extinction in the CA1 (main group effect: F(5,22) = 0.009, *p* = 0.923) (Supplemental Fig. S4B), although contextual fear recall and fear extinction induced rare c-Fos expression in the CA1. And Tat-nNOS_1–133_ did not affect fear extinction in the DG either (main group effect: F(6,21) = 0.002, *p* = 0.969) (Supplemental Fig. S4C). These results suggesting that PSD-95-nNOS coupling in the DG and CA1 is not implicated in the modulation of contextual fear extinction.

### NMDARs Activation in the CA3 Promotes Contextual Fear Extinction

**Figure 3:**
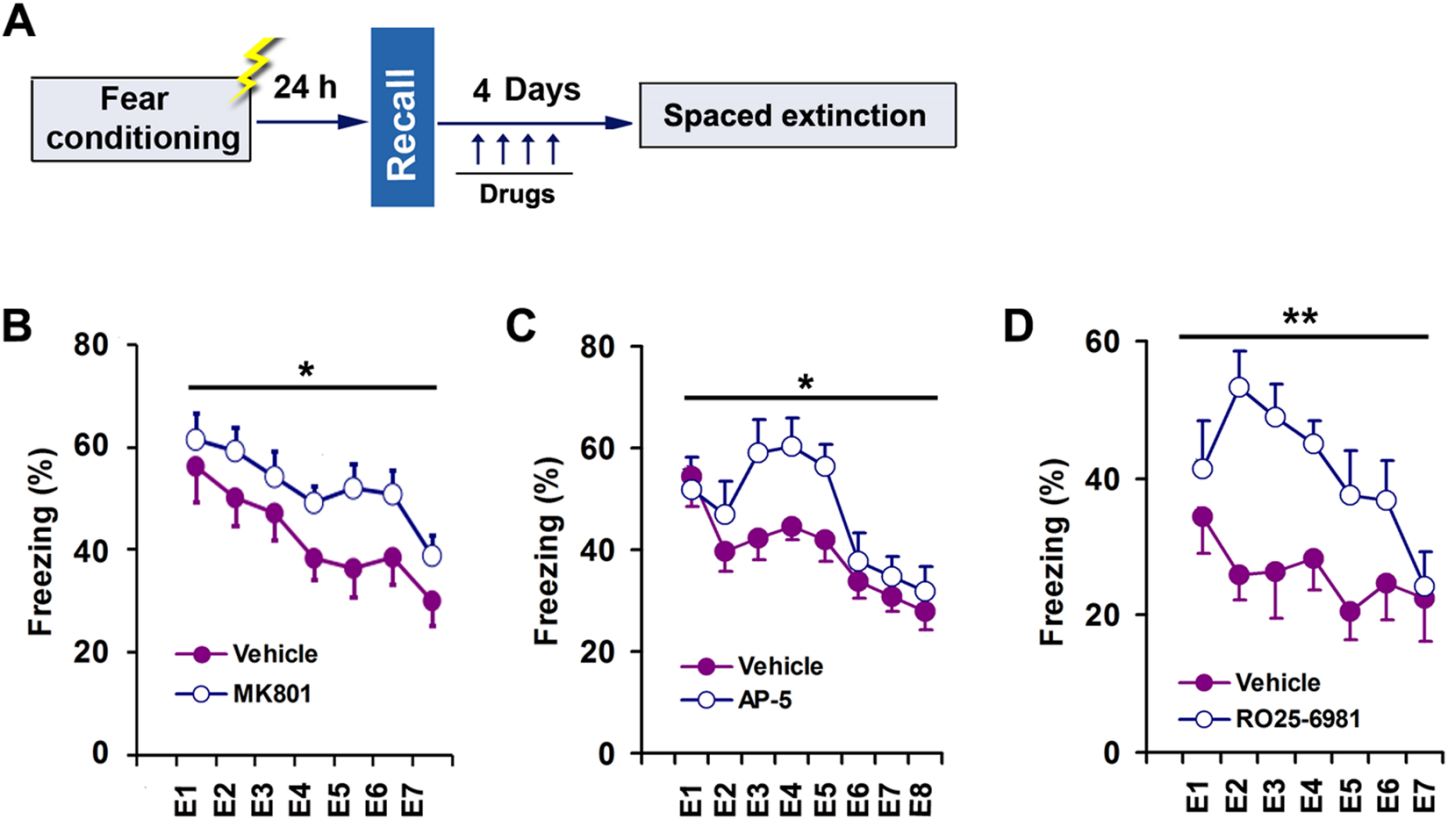
NMDARs activation in the CA3 promotes contextual fear extinction. (**A**) Design of the experiments for (B–D). (**B**) Effect of intra-CA3 MK801 on fear extinction (n = 14–15). (**C**) Effect of intra-CA3 AP-5 on fear extinction (n = 13–14). (**D**) Effect of intra-CA3 RO25-6981 on fear extinction (n = 10–11).

To determine whether NMDARs activation in the CA3 is necessary for contextual fear extinction, we observed effects of MK801, a potent non-competitive antagonist of NMDARs that use-dependently blocks the channel in the open (glutamate-bound) state. We used same contextual fear conditioning procedure as above, and 2 h after fear recall, we infused MK801 into the dorsal CA3 of conscious mice through the implanted microcannula for 4 consecutive days (Fig. 3A). As expected, mice treated with MK801 displayed significantly enhanced freezing levels, compared with vehicle (Fig. 3B), indicating reduced contextual fear extinction (main group effect: F(6,27) = 4.889, *p* = 0.036). And there is no changes in locomotor activity after treatment with MK801 in the open-field test (Supplemental Fig. S3A,D).

Next, we investigated effect of AP-5, a competitive antagonist of NMDARs, on contextual fear extinction. Two hours after fear recall, we infused AP-5 into the dorsal CA3 of conscious mice through the implanted microcannula for 4 consecutive days (Fig. 3A). Similar to MK801, AP-5 significantly reduced fear extinction (main group effect: F(7,24) = 4.286, *p* = 0.049) (Fig. 3C).

Based on the importance of GluN2B in the extinction of fear memories^10^, we specifically investigated the role of GluN2B-containing receptors in the CA3 in contextual fear extinction. Two hours after fear recall, we daily infused Ro25-6981, a selective GluN2B antagonist, into the dorsal CA3 of conscious mice through the implanted microcannula for 4 consecutive days (Fig. 3A). As MK801 and AP-5 did, Ro25-6981 significantly inhibited contextual fear extinction (main group effect: F(6,25) = 8.229, *p* = 0.008) (Fig. 3D). The treatment of RO25-6981 did not alter the basic movement in the open-field test (Supplemental Fig. S3A,E). Thus, NMDARs activation and PSD-95-nNOS coupling in the dorsal CA3 have completely different roles for contextual fear extinction.

### BDNF-TrkB Signaling Is Responsible for the Different Regulation of Fear Extinction by NMDARs Activation and PSD-95-nNOS Association

**Figure 4:**
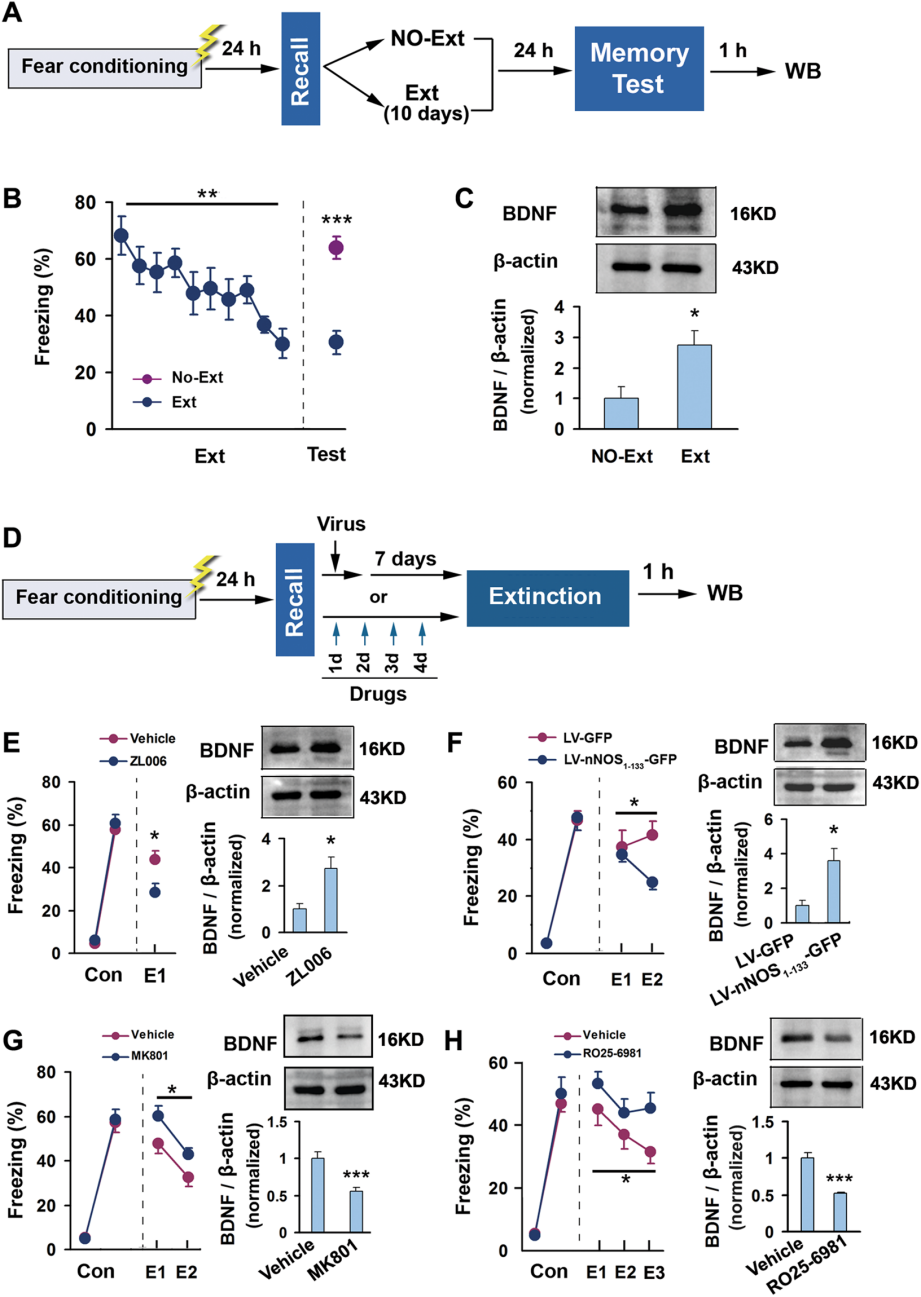
PSD-95-nNOS blockers and NMDARs antagonists oppositely regulate BDNF expression. (**A**) Design of the experiments for (B,C). (**B**) Freezing behavior measured during extinction trial and retrial of extinction memory (n = 8). (**C**) Immunoblots showing BDNF expression in the dorsal CA3 after contextual fear extinction (n = 4). (**D**) Design of the experiments for (E–H). (**E**) Effect of intra-CA3 ZL006 on fear extinction (n = 12–13) and BDNF expression in the dorsal CA3 (n = 4). (**F**) Effect of LV-nNOS_1–133_-GFP in the CA3 on fear extinction (n = 6) and BDNF expression in the dorsal CA3 (n = 3). (**G**) Effect of intra-CA3 MK801 on fear extinction (n = 12–14) and BDNF expression in the dorsal CA3 (n = 6–7). (**H**) Effect of intra-CA3 RO25-6981 on fear extinction (n = 12) and BDNF expression in the dorsal CA3 (n = 6). Ext: extinction. Con: fear conditioning.

NMDARs activation induces gene expression of BDNF^24^, a neurotrophic factor is crucial for fear extinction^16^. To investigate whether BDNF is responsible for the different role of NMDARs activation and it-mediated PSD-95-nNOS binding in contextual fear extinction, we examined BDNF expression in the dorsal CA3. After fear recall, mice were subjected to spaced extinction trial or not (Fig. 4A). We found that the mice with extinction (F(1,14) = 34.78, *p* < 0.001) showed significantly increased BDNF level (F(1,6) = 8.29, *p* = 0.028) (Fig. 4B,C). We next infused ZL006, Ro25-6981 or MK801 for 4 consecutive days, or infused LV-nNOS_1–133_-GFP on time into the dorsal CA3 through the implanted microcannula beginning 2 h after fear recall, and detected BDNF level in the dorsal hippocampus when extinctions were significantly different between groups (Fig. 4D). Disrupting PSD-95-nNOS binding in the CA3 with ZL006 (E1, F(1,23) = 6.45, *p* = 0.018) or LV-nNOS_1–133_-GFP (main group effect: F(1,10) = 5.369, p = 0.043) significantly increased BDNF expression (Fig. 4E: F(1,6) = 9.12, *p* = 0.023; Fig. 4F: F(1,4) = 10.95, *p* = 0.030) (Fig. 4E,F), whereas blocking NMDARs in the CA3 with MK801 (main group effect: F(1,22) = 4.728, p = 0.041) or Ro25–6981 (main group effect: F(1,21) = 4.606, p = 0.044) caused a significant decrease in BDNF level (Fig. 4G: F(1,11) = 37.76, *p* < 0.001; Fig. 4H: F(1,10) = 37.40, *p* < 0.001) (Fig. 4G,H). Thus, PSD-95-nNOS blockers and NMDARs antagonists oppositely regulate BDNF expression.

**Figure 5:**
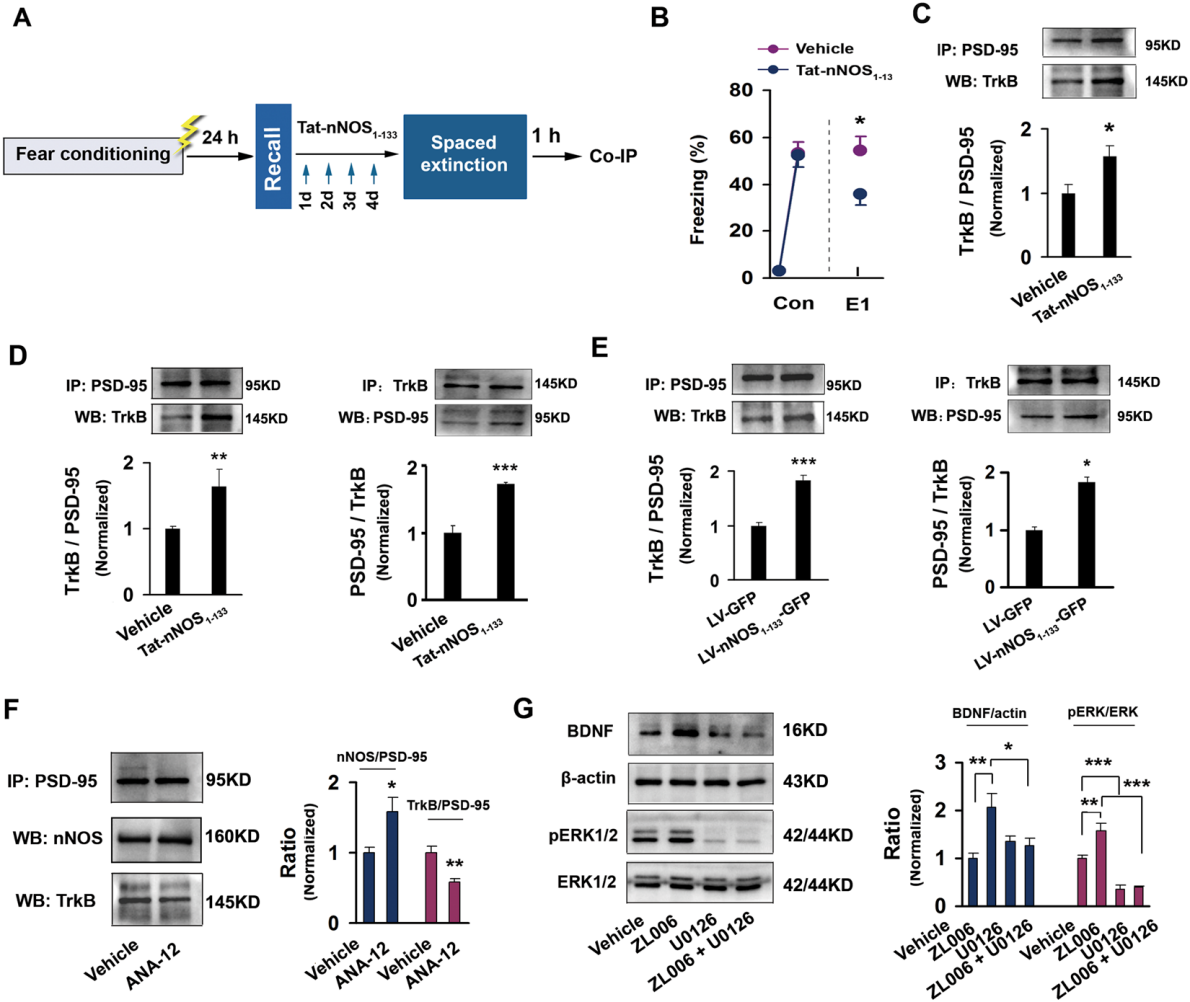
Mechanisms underlying the role of PSD-95-nNOS in regulating BDNF-TrkB signalling. (**A**) Design of the experiments for (B,C). (**B**) Effect of intra-CA3 Tat-nNOS_1–133_ on fear extinction (n = 12–13). (**C**) PSD-95-TrkB complex level in the dorsal CA3 after extinction (n = 4–6). (**D**,**E**) PSD-95-TrkB complex level in the cultured hippocampal neurons treated by Tat-nNOS_1–133_ ((**D**) Left: n = 6–7; Right: n = 6–7) or infected with LV- nNOS_1–133_-GFP ((E) Left: n = 9; Right: n = 7–9). (**F**) Effect of intra-CA3 ANA-12 on PSD-95-nNOS and PSD-95-TrkB complex levels in the dorsal CA3 (PSD-95-nNOS: n = 6; PSD-95-TrkB). (**G**) ERK inhibitor reverses the effect of ZL006 on BDNF expression and ERK phosphorylation (n = 6).

The activation of BDNF-TrkB signaling by NMDARs recruits more PSD-95 to synapses via the association of TrkB with PSD-95^18,25^ and supports extinction learning^26^, which raises a possibility that uncoupling PSD-95-nNOS leads to an increase in association of TrkB with PSD-95 and thereby enhances BDNF-mediated extinction. To address this notion, we infused Tat-nNOS_1–133_ or vehicle through microcannula into the dorsal CA3 of mice for 4 consecutive days beginning 2 h after fear recall, and detected PSD-95-TrkB complex levels in the dorsal CA3 when extinctions were significantly different between groups (E1, F(1,23) = 6.65, *p* = 0.017) (Fig. 5A,B). Disrupting PSD-95-nNOS binding in the CA3 significantly increased association of PSD-95 with TrkB (F(1,8) = 9.59, *p* = 0.015) (Fig. 5C). To further address this, cultured hippocampal neurons were exposed to Tat-nNOS_1–133_ for 24 h or LV- nNOS_1–133_-GFP for 7 d. Similar with the findings in *in vivo*, blocking PSD-95-nNOS in *in vitro* significantly increased PSD-95-TrkB complex levels also (Fig. 5D, Left: F(1,11) = 10.53, *p* = 0.008; Right: F(1,11) = 44.62, p < 0.001; Fig. 5E, Left: F(1,16) = 33.22, p < 0.001; Right: F(1,14) = 8.42, *p* = 0.012) (Fig. 5D,E). Next, we infused ANA-12, a TrkB receptor antagonist, into the dorsal CA3 of mice, and 24 h later, detected PSD-95-nNOS and PSD-95-TrkB complexes. The drug significantly decreased PSD-95-TrkB complex (F(1,10) = 20.78, *p* = 0.001) and increased PSD-95-nNOS complex (F(1,10) = 8.47, *p* = 0.016) (Fig. 5F). Collectively, these findings suggest a competition between nNOS and TrkB for binding to PSD-95. Thus, NMDARs activation and PSD-95-nNOS binding have distinct role in regulating BDNF-TrkB signaling.

To address this, we treated cultured hippocampal neurons with ZL006, U0126 (an ERK inhibitor) or combination of two drugs for 24 h, and measured BDNF and phosporylated ERK (pERK) levels. Indeed, blocking PSD-95-nNOS by ZL006 significantly increased pERK level and BDNF expression, ERK inhibitor reversed the effects of ZL006 (BDNF/β-actin: F(3,20) = 6.09, vehicle *vs* ZL006, *p* = 0.006; ZL006 vs ZL006 + U0126, *p* = 0.048. pERK/ERK: n = 6, F(3,20) = 34.97, vehicle *vs* ZL006, *p* = 0.005; vehicle vs U0126, *p* = 0.001; ZL006 vs ZL006 + U0126, p < 0.001) (Fig. 5G). NMDARs activation enhances BDNF production through ERK phosporylation^27^. Therefore, NMDARs activation and PSD-95-nNOS coupling may oppositely regulate ERK phosphorylation and thereby leading to a bidirectional regulation of BDNF expression.

**Figure 6:**
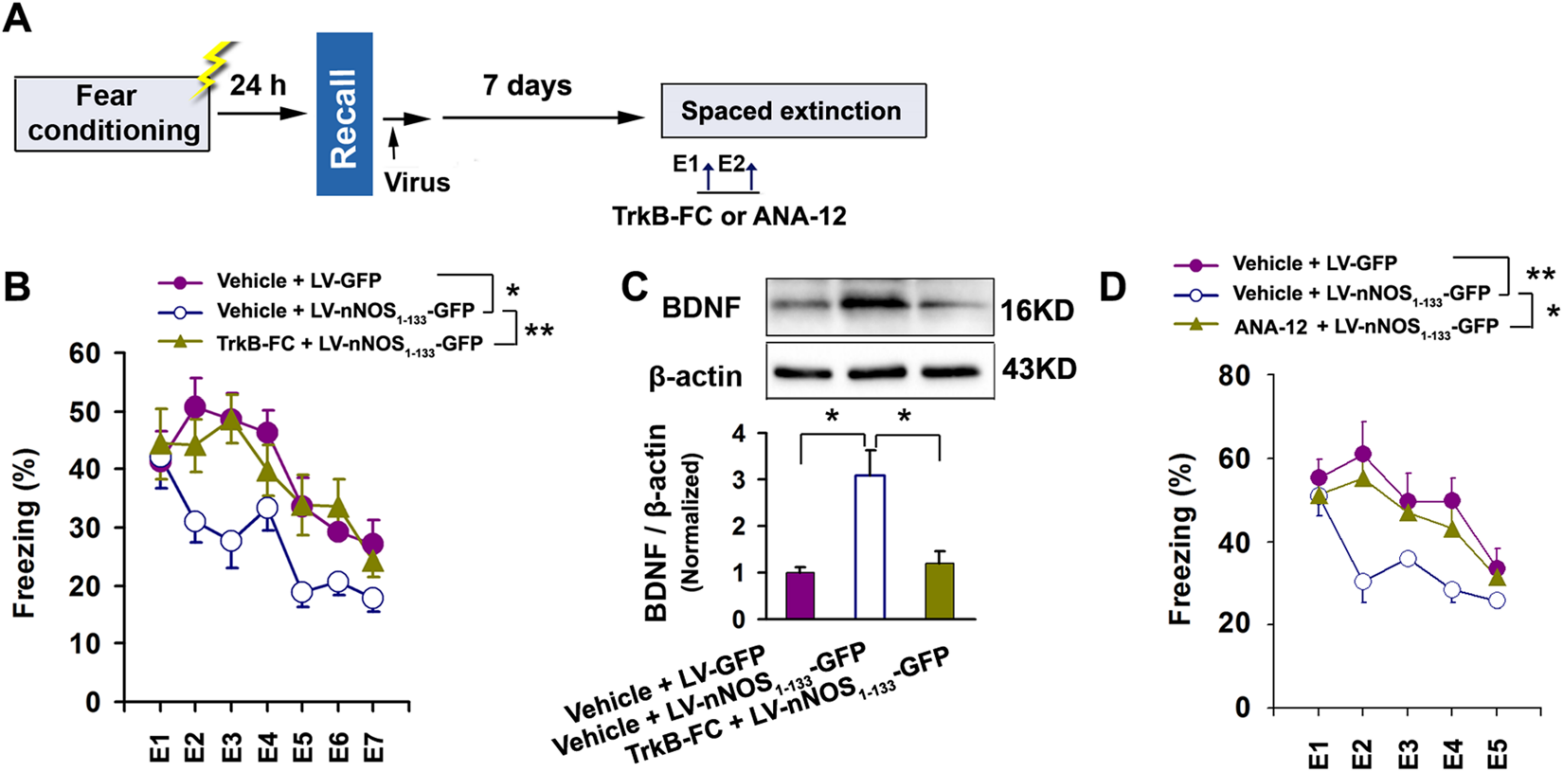
The role of PSD-95-nNOS in regulating contextual fear extinction is BDNF-dependent. (**A**) Design of the experiments for (B–D). (**B**) BDNF scavenger TrkB-FC reversed the effect of LV-nNOS_1–133_-GFP on fear extinction (n = 13). (**C**) BDNF scavenger TrkB-FC reversed the effect of LV-nNOS_1–133_-GFP on BDNF expression (n = 4–5). (**D**) TrkB receptor antagonist ANA-12 reversed the effect of LV-nNOS_1–133_-GFP on fear extinction (n = 11).

To determine whether BDNF is requirement for the role of PSD-95-nNOS in contextual fear extinction, we infused LV-nNOS_1–133_-GFP or its control LV-GFP into the dorsal CA3 of mice through the implanted microcannula after fear recall. Seven days later, the mice were subjected to contextual fear extinction for 7 consecutive days (E1-E7), and we infused TrkB-FC, a BDNF scavenger, into the dorsal CA3 after E1 and E2 to remove local BDNF (Fig. 6A). Fear levels after recall were similar between groups. LV-nNOS_1–133_-GFP significantly reduced freezing behavior (F(2,36) = 6.15, *p* = 0.008, *p* = 0.018) and up-regulated BDNF expression in the dorsal CA3, compared to LV-GFP, and TrkB-FC completely reversed the effects of LV-nNOS_1–133_-GFP (main group effect: F(2,32) = 5.933, *p* = 0.006; *post hoc* comparisons, vehicle + LV-GFP *vs* vehicle + LV-nNOS_1–133_-GFP, *p* = 0.027; vehicle + LV-nNOS_1–133_-GFP *vs* TrkB-FC + LV-nNOS_1–133_-GFP, *p* = 0.009) (Fig. 6B,C). Next, we infused ANA-12, a TrkB receptor antagonist, into the dorsal CA3 after E1 and E2 to block BDNF receptor (Fig. 6A). With similar fear levels between groups after recall, ANA-12 completely reversed the effect of LV-nNOS_1–133_-GFP on contextual fear extinction (main group effect: F(2,30) = 8.580, *p* = 0.004; *post hoc* comparisons, vehicle + LV-GFP *vs* vehicle + LV-nNOS_1–133_-GFP, *p* = 0.001; vehicle + LV-nNOS_1–133_-GFP *vs* ANA-12 + LV-nNOS_1–133_-GFP, *p* = 0.017) (Fig. 6D). Together, these data suggest that the role of PSD-95-nNOS on extinction is BDNF-dependent.

## Discussion

Impairments in fear extinction are thought to be central to the psychopathology of PTSD. Crucial role of NMDARs in the limbic system for fear extinction^5–7^, especially, the role of GluN2B-containing NMDARs^10^, offers an inviting strategy to treat PTSD by activating NMDARs. However, NMDARs activation has a completely opposite effect on other affective processes, such as depression- and anxiety-related behaviors^28–30^. Because PTSD is often complicated by general anxiety and depressed mood^31^, the treatment targeting on NMDARs for PTSD may pose a challenge, render their use problematic. Activation of GluN2B-containing NMDARs induces the interaction of nNOS with PSD-95^12,13^. Using different behavioral protocols, we demonstrated that dissociation of nNOS from PSD-95 in the CA3 promoted contextual fear extinction, while NMDARs antagonists impaired contextual fear extinction. To our knowledge, this is the first report revealing the significance of PSD-95-nNOS coupling for fear extinction, and showing opposite roles of NMDARs activation and NMDARs-mediated PSD-95-nNOS coupling in regulating extinction. Because inhibitors of PSD-95-nNOS interaction produce antidepressant and anxiolytic effect without unwanted side effects associated with NMDARs^13,32,33^, PSD-95-nNOS could be a valuable target for the treatment of PTSD.

The hippocampus is a crucial part of the neuronal circuit mediating fear extinction. Pharmacological inhibition of hippocampal Cdk5 activity facilitates extinction in the contextual fear-conditioning procedure^34^. DG was reported to be involved in fear extinction learning and adult-born neurons in the DG are integral for the maintenance of remote contextual fear memory^35,36^. Moreover, inhibition of Rac1 activity in the CA1 has been shown to impair massed extinction of contextual fear^37^. Here, we provide strong evidence that CA3 is an important hippocampal subregion playing crucial roles in fear extinction.

Because of the extensive recurrent collateral system connecting pyramidal cells of the CA3 area of hippocampus, CA3 is thought to promote the flexible formation and reorganization of information-coding ensembles and serve as an associative memory network^38–40^. Within the hippocampus, the NMDARs presumably participate in plastic synaptic events in the CA1 and CA3, the principal pyramidal cell fields^39^. However, the hippocampal subfields CA1 and CA3 are functionally segregated^41^. Indeed, after contextual fear extinction, immediate early gene c-Fos is abundantly expressed in the CA3 but not in the CA1. Our findings that uncoupling PSD-95-nNOS in the CA3 but not in the CA1 facilitates contextual fear extinction could be a presentation of function difference between CA1 and CA3. Although the number of c-Fos-positive cells in the CA1 was rarely increased after contextual fear recall and contextual fear extinction, this change may be due to the projection from CA3 c-Fos positive cells. However, CA3 and CA1 have different efferent pathway. CA3 projects to the septofimbrial nucleus, throughout the rostral lateral septum, the dorsal medial septum, and the dorsal-most portions of the vertical limb of the diagonal band of Broca. CA1 projects to the septofimbrial nucleus, the ventral portions of the medial septum, as well as the horizontal limb of the diagonal band of Broca^42,43^. These differential targets of CA3 and CA1 subcortical efferents suggest that there may be functional heterogeneity in the classical fear conditioning^44^.

For the contextual memory, CA3 NMDARs are required for the rapid formation of a salient contextual representation^45^, whereas DG NMDARs mediate rapid pattern separation in the hippocampal network^46^, which may explain our results that uncoupling PSD-95-nNOS in the CA3 but not in the DG promoted contextual fear extinction.

BDNF in the limbic system are crucial for fear extinction^3,16,47^. NMDARs activation not only induces BDNF gene expression in hippocampal neurons^24^, but also facilitates BDNF-TrkB signaling to recruit PSD-95 to synapses and promotes extinction learning^18,25,26^. In contrast, we found that disrupting the association of nNOS with PSD-95, downstream of NMDARs activation, up-regulated BDNF expression and association of BDNF-TrkB signaling with PSD-95. More importantly, BDNF scavenger and TrkB receptor antagonist abolished the effect of LV-nNOS_1–133_-GFP on fear extinction. Thus, PSD-95-nNOS interaction regulates contextual fear extinction via BDNF-TrkB signaling. Furthermore, we showed that contextual fear extinction induced a shift from PSD-95-nNOS to PSD-95-TrkB coupling. Therefore, different BDNF-TrkB signaling in the CA3 may explain different roles of NMDARs activation and NMDARs-mediated PSD-95-nNOS coupling in regulating extinction.

It is generally believed that synaptic NMDAR conveys the synaptic activity-driven activation of the survival-signaling protein ERK leading to the activation of the transcription factor CREB and the production of the survival-promoting protein BDNF^27^. Thus, ERK-BDNF signaling pathway may account for the role of NMDARs activation in fear extinction. PSD-95-nNOS interaction facilitates NO production^13^. nNOS-derived NO is a potent inhibitor of Ca^2+^-mediated ERK activation^20^. Here, we found that uncoupling PSD-95-nNOS increased ERK phosphorylation and ERK inhibitor reversed the effect of ZL006 and Tat-nNOS_1–133_ on BDNF expression. Thus, PSD-95-nNOS interaction may down-regulate BDNF expression via inhibiting ERK activation. However, we can not exclude the possibility that PSD-95-nNOS interaction may have other way to regulate BDNF, such as histone deacetylases (HDACs)-mediated epigenetic modification. HDACs play a negative role in histone H3 and H4 acetylation of BDNF promoters, thereby influence BDNF expression^48^. It has been reported that nitrosylation of HDAC2 is implicated in the regulation of fear memory^15^. Moreover, our previous study showed that NO up-regulates HDAC2 level and activity^14^. Thus, further investigation will be required to test whether HDACs are implicated in the regulation of BDNF expression and consequent fear extinction by PSD-95-nNOS coupling.

Apart from decreased level of PSD95-nNOS coupling (F(1,8) = 9.88, *p* = 0.010) (Supplemental Fig. S5), directly blocking NMDARs also affect other signaling pathway via Ca^2+^ influx, such as BDNF-TrkB signalling. But disassociating PSD-95-nNOS coupling, the downstream signalling of NMDAR activation, has no effect on Ca^2+^ influx. Thus, NMDARs activation and NMDARs-mediated PSD-95-nNOS coupling may play a different role in modulating contextual fear extinction.

**Figure 7:**
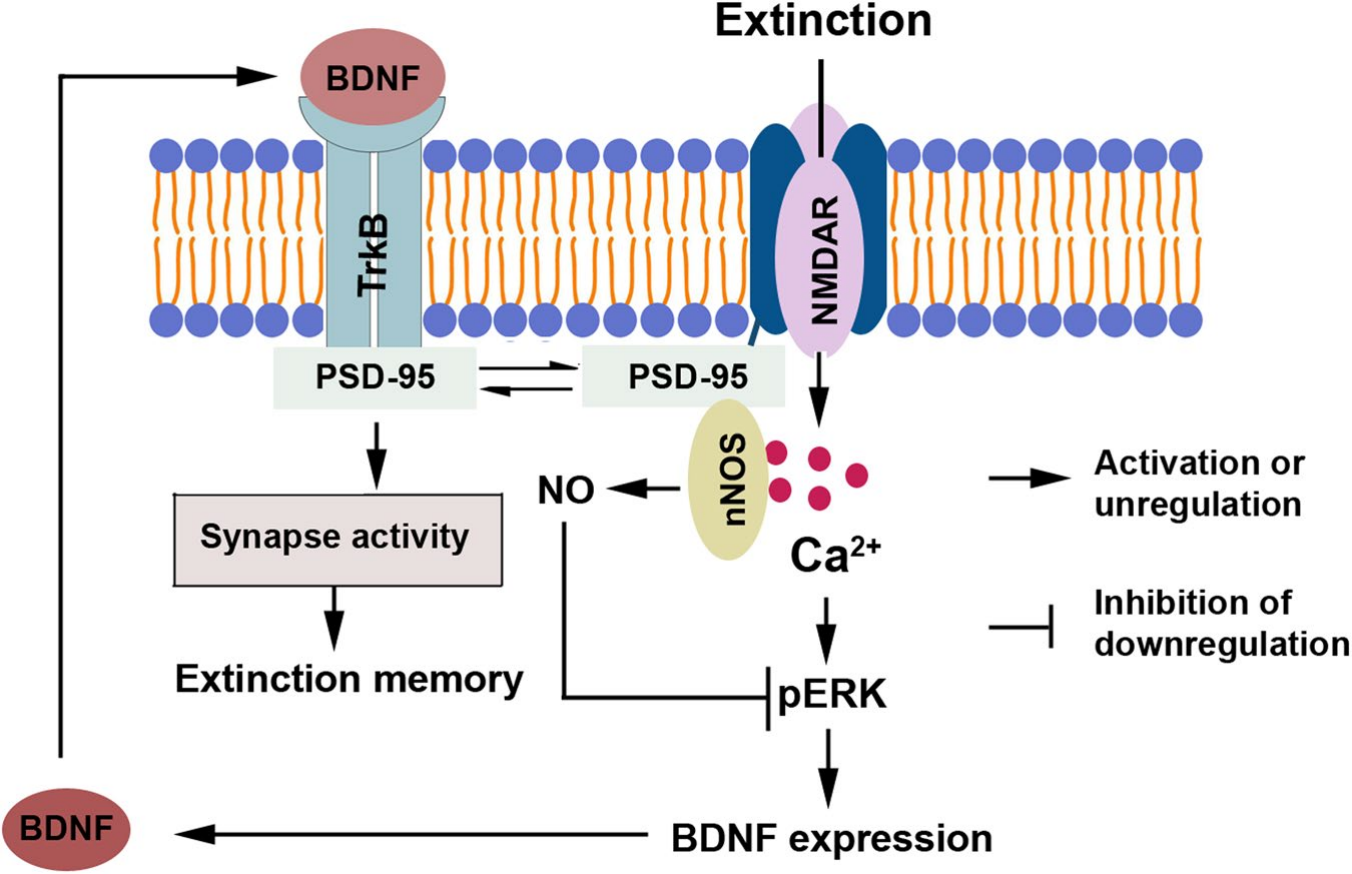
A model of signaling pathway whereby NMDARs activation and it-mediated PSD-95-nNOS interaction differently regulate contextual fear extinction.

Collectively, NMDARs activation up-regulates BDNF expression via Ca^2+^-mediated ERK phosphorylation and enhances the association of BDNF-TrkB signaling with PSD-95, subsequently enhances synapse activity, thereby promotes contextual fear extinction. NMDARs-mediated PSD-95-nNOS interaction weakens the role of NMDARs activation via reducing ERK phosphorylation and BDNF-TrkB signaling with PSD-95 (Fig. 7). Thus, blocking PSD-95-nNOS and activating NMDARs produce similar effect on fear extinction. Different from NMDARs activation, however, uncoupling PSD-95-nNOS is beneficial to both fear extinction and mood. Our present work provides an important addition to the knowledge of modulation of extinction, and suggests that PSD-95-nNOS could be a valuable target for the treatment of PTSD.

## Methods and Materials

Detailed and extended data are available in the Supplement.

### Animals

Young adult (6- to 7-week-old) male homozygous nNOS-deficient mice (B6;129S4*-Nos1*^*tm1Plh*^, knockout, stock number: 002633) and their wild-type controls of similar genetic background (B6129SF2, WT) (both from Jackson Laboratories, maintained at Model Animal Research Center of Nanjing University, Nanjing, China), and young adult (6–8 weeks) male C57BL/6 mice were used in this study. All procedures were approved by the Institutional Animal Care and Use Committee of Nanjing Medical University. And all experiments were performed in accordance with the approved guidelines and regulations. Every effort was made to minimize the number of animals used and their suffering.

### Surgical Procedures and Drug Infusions

For intra-hippocampus infusions, Guide cannulae (26 gauge; Plastics One, RWD Life Science) were unilaterally implanted 1.5 mm above the dorsal hippocampus using coordinates for the CA3: −1.7 mm AP; +1.9 mm ML; −1.9 mm DV; for the CA1: −1.7 mm AP; +1.3 mm ML; −1.8 mm DV; for the DG: −1.7 mm AP; +1.0 mm ML; −2.1 mm DV. Drug infusions at a rate of 0.06 μl/min (Harvard Apparatus, Holliston, MA) included ZL006, Tat-nNOS_1–133_, LV-nNOS_1–133_ -GFP, MK801, AP-5, RO25-6981, TrkB-FC and ANA-12.

### Contextual Fear Conditioning and Extinction

Extinction and reminder shock procedures were carried out as described previously^34^. In brief, contextual fear conditioning was performed with a computerized fear conditioning system (CSI Systems). The freezing level was quantified from digitized video images using commercially available software (Freezescan, Clever Systems, Reston, VA). Animals were allowed to explore the training cage for 3 min followed by a mild electric shock (2 s, 0.7 mA). Context-dependent freezing, defined as the absence of movements other than those required for breathing, was assessed 24 h later by re-exposing the mice for 3 min into the conditioning context. Spaced extinction of contextual fear was performed on consecutive days, consisting of re-exposure to the training context in a non-reinforced manner for 3 min. An extinction protocol is composed by time of each extinction trial and the number of extinction trial. A complete extinction is indicated by an appearance of extinction bottom. However, the dynamics of fear extinction can vary among experiments. In other words, the number of extinction trials to get extinction bottom could be different. In our experiments, the number of extinction trials varied from 5 to 10 days, although time of each extinction trial was same. In the experiment about extinction failure and extinction success, “Extinction Failure” and “Extinction Success” were established based on the freezing values in the last extinction trial. Freezing level higher than 65% of the acquisition level was defined “Extinction Failure” and lower than 45% of the acquisition level was defined “Extinction Success”.

### Cell Cultures, Recombinant Lentivirus and Fusion Peptide

Culture of hippocampal neurons, the production of recombinant lentivirus, LV-nNOS_1–133_-GFP or its control LV-GFP, and the preparations of fusion peptide, Tat-nNOS-N_1–133_, were performed as we previously reported^13,14,49^ using the procedures detailed in the Supplement.

### Western Blot Analysis and Coimmunoprecipitation

The samples of CA3 in the dorsal hippocampus were obtained as follows^50^: the brains were removed and blocked rapidly over ice into coronal sections. Serial hippocampal sections (200 μm) were made on a vibratome (Leica) in a bath of pre-cold PBS. Then the CA3 of cannulas-implanted side was obtained in the first six pieces of hippocampal sections under a dissecting microscope and placed in the pre-cooling RIPA lysate. Samples from cultured neurons were prepared as described by our previous studies^49^. Western blot analysis and coimmunoprecipitation used the procedures detailed in the Supplement.

### Statistical analysis

In behavioral experiments, freezing data were analyzed with a repeated-measures analysis of variance (ANOVA) followed by Tukey’s *post hoc* test^47^. In some cases, with only one factor (Extinction memory test in Fig. 1B) in the experiment, we employed one-way ANOVA (one factor) followed by Tukey’s *post hoc* test to compare means from two groups at the same time. Student’s t-tests were used for statistical comparison between two groups. Data were presented as the mean ± SEM, and *p* < 0.05 was considered statistically significant. Investigators were blind to the group allocation when assessing the outcome. For the effects of drugs on fear extinction, animals were randomly assigned into different groups immediately after fear recall.

## Electronic supplementary material

supplementary information
