# Supplementary material for: PSD-95-nNOS Coupling Regulates Contextual Fear Extinction in the Dorsal CA3
Source: Sci Rep. 2018 Aug 24;8:12775. doi: 10.1038/s41598-018-30899-4 (PMC6109109; doi:10.1038/s41598-018-30899-4)
Supplement: Supplementary file 1 — supplementary information [file 41598_2018_30899_MOESM1_ESM.doc]

**PSD-95-nNOS Coupling Regulates** **Contextual Fear Extinction in the Dorsal CA3**

Cheng-Yun Cai^1,2,*^, Chen Chen^1,2,*^, Ying Zhou^1,2,*^, Zhou Han^1,2^,, Cheng Qin^1,2^,, Bo Cao^1,2^,, Yan Tao^1,2^,, Xin-Lan Bian^1,2^,, Yu-Hui Lin^1,2^,, Lei Chang^1,2^,, Hai-Yin Wu^1,2^,, Chun-Xia Luo^1,2^,, Dong-Ya Zhu^1,2,3,#^

^1^Institution of Stem Cells and Neuroregeneration, Nanjing Medical University, Nanjing 211166, People’s Republic of China.

^2^Departments of Pharmacology, School of Pharmacy, Nanjing Medical University, Nanjing 211166, People’s Republic of China.

^3^The key laboratory of human functional genomics of Jiangsu Province, Nanjing 211166, People’s Republic of China.

^*^These authors contributed equally to this work.

^#^Address correspondence to Dong-Ya Zhu, Ph.D., Institution of Stem Cells and Neuroregeneration, Nanjing Medical University, 101 Longmian Avenue, Jiangning District, Nanjing 211166, P.R. China. Email: [dyzhu@njmu.edu.cn](mailto:dyzhu@njmu.edu.cn).

*Supplemental Information*

**Supplementary Methods and Materials**

**Surgical Procedures**

Adult mice were anesthetized with 1-2% isoflurane and then placed in a stereotaxic apparatus. Guide cannulae (26 gauge; Plastics One, RWD Life Science) were unilaterally implanted 1.5 mm above the dorsal CA3 area of the hippocampus (coordinates: -1.7 mm AP; +1.9 mm ML; -1.9 mm DV), the dorsal CA1 area of the hippocampus (coordinates: -1.7 mm AP; +1.3 mm ML;-1.8 mm DV), or the dorsal DG area of the hippocampus (coordinates: -1.7mm AP; +1.0 mm ML; -2.1 mm DV). The cannulas were anchored to the skull with stainless-steel screws and dental cement. A stainless-steel stylet blocker was inserted into each cannula to keep it patent and prevent infection. The mice were allowed to recover for at least 1 week after surgery.

**Drugs**

ZL006 was synthesized and Tat-nNOS_1-133_ was prepared in our laboratory. MK801, AP-5, RO25-6981 and U0126 were purchased from Sigma (St Louis, MO). TrkB-FC and ANA-12 were purchased from R&D system. ZL006 was dissolved in 0.8% sodium bicarbonate solution, Tat-nNOS_1-133_, MK801, AP-5, RO25-6981, TrkB-FC and ANA-12 were dissolved in 0.9% saline, and U0126 was dissolved in DMSO. The concentration of ZL006 and Tat-nNOS_1-133_ was 10 μM and 50 nM respectively. The concentrations of other drugs were based on previous studies: 3 mM MK801^1, 2^, 5 mM AP-5^1, 3^, 3 μM RO25-6981^4^, 10 μM U0126^5^, 1.0 mg/ml TrkB-FC^6^ and 10 μM ANA-12^7^. For drug infusions, obturators were removed and injectors were placed into the guide cannulas. Injector tips extended 0.6 mm, 0.3 mm or 0.4 mm beyond the guide cannula. One day before testing, mice were habituated for handling and injectors were passed through the cannula (without infusion). Each infusion volume was 1 μl per side infused at a rate of 0.06 μl/min. Unilateral microinfusions were made through 31 gauge injection cannulae that was connected to a 10 μl microsyringe mounted in the microinfusion pump (Harvard Apparatus), and to control the diffusion of the drug solutions, we divided the volume of 1 µL into four times for injection. The volume of each injection was 0.25 µL, and every time the spread of drugs infusion was 1 nl/s and an additional 2 min was given for drug diffusion. Animals were returned to their home cage after infusion.

**Culture of Hippocampal Neurons.**

Primary hippocampal neurons were isolated and cultured as described previously^8^ with some modifications. Hippocampi of embryo day 15 (E15) mice were removed and placed in Hanks’ balanced salt solution (HBSS) without Ca^2+^ and Mg^2+^ (Gibco BRL, Grand Island, NY) containing 1 mM sodium pyruvate and 10 mM HEPES. Then the hippocampal tissues were dissociated in HBSS solution containing 0.125% trypsin solution for 10 min at 37 °C. Subsequently, tissues were triturated by repeated passage through a constricted Pasteur pipette. The digestion was stopped with DMEM along with 10% heat-inactivated fetal bovine serum. The dispersed tissues were allowed to settle for 3 min. The supernatant was transferred to a fresh tube and centrifuged at 2500 rpm for 5 min. The pellet was re-suspended in a neuron-defined culture medium, serum-free neurobasal medium (Gibco), supplemented with B-27, 0.5 mM L-glutamine, 20 IU/ml penicillin and 20 IU/ml streptomycin. The cells were then plated onto 3.5 cm or 6.0 cm diameter dish coated with poly-D-lysine (100 μ g/ml) at 1.0 × 10^5^ /cm^2^ for western analysis and 1.5 × 10^5^ /cm^2^ for coimmunoprecipitation. Cell cultures were kept in a humidified atmosphere of 95% air and 5% CO_2_ at 37 °C. Half of the medium was replaced with fresh medium without glutamate every 2–3 days. The purity of neuronal cultures was determined by immunofluorescence using staining with antibody against β-III-tubulin (1:200; Millipore), and the nuclei were stained for 15 min with Hoechst 33258 (1 μg/ml in PBS). The purity of the neurons used to experiments was ~95%.

**Recombinant Virus Production and Infection**

The recombinant lentivirus, LV-nNOS_1–133_ -GFP or its control LV-GFP, was generated as we previously reported^9^. LV-nNOS_1–133_ -GFP selectively expresses N-terminal amino acid residues 1–133 of nNOS (nNOS-N_1–133_), a region crucial for nNOS–PSD-95 interaction. Cultured neurons were infected with LV-nNOS_1–133_ -GFP or LV-GFP containing 1.0 × 10^9^ transduction units/ml at d 4 in vitro [multiplicity of infection (MOI)=2.5]. The medium was half changed 8 h later and fully changed 24 h later. For lentivirus infection in vivo, Intracranial microinjection of 1 μl of virus suspension to hippocampus was carried out. Seven days after lentivirus infusion, the mice were subjected to contextual fear extinction.

**Tat-nNOS_1–133_ Preparation**

The recombinant fusion protein of Tat-nNOS_1–133_ was generated as we previously reported^10^. Gene fragment encoding nNOS_1–133_ was assembled by RT-PCR, and Tat was added to its 5 end by add-PCR. The gene fragment was inserted into a T7 RNA polymerase-based expression system pET28a vector (Novagen). We named it pET28a-Tat-nNOS_1–133_. The pET28a-Tat-nNOS_1–133_ was transformed into E. coli strain BL21 cultures, which were grown in LB medium containing kanamycin overnight at 37°C, until the OD 600 of 0.8 was reached, and then they were cultured for 6 h by adding isopropyl β- D -thiogalactoside (0.7 mM ). The recombinant fusion protein of Tat-nNOS_1–133_ was expressed into inclusion bodies. Bacteria were re-suspended with lysis buffer (50 mM Tris-HCl, 10 mM EDTA, 100 mM NaCl, pH 8.0) and subsequently lysed with ultrasonication. Most of the other bacterial proteins were eliminated during the early steps of washing the resulting pellet with wash buffer (50 mM Tris-HCl, 0.5% Triton X-100, pH 8.0) and 2 M urea. Inclusion bodies were solubilized by 8 M urea, and Tat-nNOS_1–133_ was refolded by dialysis with a renaturation buffer (50 mM Tris-HCl, 0.5 mM EDTA, and 50 mM NaCl, 8% glycerol, pH 8.0) at 4 °C. After centrifugation at 12,000 g for 10 min, supernatant containing refolded protein was loaded on a DEAE-52 column, and Tat-nNOS_1–133_ was eluted by gradient elution (50 mM Tris-HCl, 0.5 mM EDTA, 0~200 mM NaCl, pH 8.0). The protein fractions corresponding to Tat-nNOS_1–133_ were collected and analyzed using SDS-PAGE. Tat-nNOS_1–133_ was placed into PBS and concentrated with Amicon Ultra-15 Centrifugal Filter Units.

**Western Blot Analysis**

The samples of CA3 in the dorsal hippocampus were obtained as follows^9^: the brains were removed and blocked rapidly over ice into coronal sections. Serial hippocampal sections (200 μm) were made on a vibratome (Leica) in a bath of pre-cold PBS. Then the CA3 of cannulas-implanted side was obtained in the first six pieces of hippocampal sections under a dissecting microscope and placed in the pre-cooling RIPA lysate (containing 150 mM NaCl, 1 mM EDTA-Na, 1%NP-40, 0.02% sodium azide, 0.1% SDS, 0.5% sodium deoxycholate, 1%PMSF, 1‰ aprotinin, 1‰ leupeptin, and 0.5‰ pepstatin A). These samples were kept frozen at 80 °C until western blot assay. Samples from cultured neurons were prepared as described by our previous studies^6^. The samples containing equivalent amounts of protein (20 μg) were applied to 10% acrylamide denaturing gels (SDS-PAGE). The separated proteins were transferred onto polyvinylidene difluoride membranes overnight at 4 °C. Blotting membranes were incubated with blocking solution (5% nonfat dried milk powder dissolved in TBST buffer (pH 7.5, 10 mM Tris-HCl, 150 mM NaCl, and 0.1% Tween 20)) for 1 h at room temperature, washed three times, and then were incubated with rabbit anti-PSD95 (1:2000; Cell Signaling Technology Cat# 3450S), rabbit anti-nNOS (1:1000; Thermo Fisher Scientific), rabbit anti-BDNF (1:1000; Thermo Fisher Scientific), mouse anti-TrkB (1:1000; BD Biosciences), rabbit anti-ERK (L352;1:2000; Bioworld Technology) or rabbit anti-p-ERK (1:1000; Bioworld Technology) in TBST overnight at 4 °C. Internal control was performed using mouse anti-GAPDH (1:8000; KangChen Biotech) or rabbit anti-β-actin (1:2000; Bioss). After several washes with TBST buffer, appropriate horseradish peroxidase-linked secondary antibodies were used for detection by enhanced chemiluminescence (Pierce). The films were scanned and densitometry was performed using the ‘Quantity One’ image software (Bio-Rad). The relative level of the protein was quantified from the scanned films.

**Coimmunoprecipitation**

The samples of dorsal hippocampus were obtained as follows^11^: the brains were removed and blocked rapidly over ice into coronal sections. Serial hippocampal sections (400 μm) were made on a vibratome (Leica) in a bath of pre-cold PBS. Then the cannulas-implanted side was obtained in the first three pieces of hippocampal sections and placed in the pre-cooling RIPA lysate (containing 150 mM NaCl, 1 mM EDTA-Na, 1% NP-40, 0.02% sodium azide, 0.1% SDS, 0.5% sodium deoxycholate, 1% PMSF, 1‰ aprotinin, 1‰ leupeptin, and 0.5‰ pepstatin A). These samples were kept frozen at 80 °C. Lysis and coimmunoprecipitation of cultures was performed as we described previously^7^ with some modification. Cultured neurons or dorsal hippocampal tissues were lysed in 50 mM Tris-HCl, pH 7.4, buffer containing 150 mM NaCl, 1 mM EDTA-Na, 1%NP-40, 0.02% sodium azide, 0.1% SDS, 0.5% sodium deoxycholate, 1% PMSF, 1‰ aprotinin, 1‰ leupeptin, and 0.5‰ pepstatin A. The lysates were centrifuged at 12,000 g for 15 min at 4 °C. The supernatant (200 μl) was preincubated at 4 °C with 4 μl of caputure antibody for 6 hours, and then add the antibody-antigen complex to the washed Protein G Magnetic beads overnight at 4 °C. The capture antibody were rabbit anti-nNOS (1:100, BD Biosciences), rabbit anti-TrkB (1:50, Santa Cruz), rabbit anti-PSD-95 (1:100, Cell Signaling Technology). Immune complexes were isolated by the magnetic stand, washed three times with 0.05 M HEPES buffer, pH 7.1, containing 0.15% Triton X-100, 0.15 M NaCl, and 0.1 × 10^-3^ M sodium orthovanadate; and bound proteins were eluted by heating at 100 °C in loading buffer. Proteins were analyzed by immunoblotting using mouse anti-PSD-95 (1:4000; Abcam), rabbit anti-nNOS (1:500; Thermo Fisher Scientific) or mouse anti-TrkB (1:1000; BD Biosciences).

**Immunofluorescence**

After behavior testing, mice were deeply anesthetized with ketamine (150 mg/ kg, respectively). Animals were perfused transcardially with 200 ml 0.05 M sodium phosphate (pH 7.4) containing 0.9% NaCl, followed by 300 ml 4% paraformaldehyde in 0.05 M sodium phosphate (pH 7.4, containing 0.9% NaCl). Brains were removed and post fixed overnight in the same solution. Serial hippocampal sections (40 μm) were made on a vibratome (Leica) in a bath of PBS. For immunocytochemistry, slices were permeabilized with 0.1% Triton X-100, blocked and incubated overnight with 0.1% Triton X-100/10% fetal bovine serum in PBS containing rabbit anti-c-Fos antibody (1:500; Synaptic Systems). Subsequently, the sections were incubated with secondary antibodies goat anti-rabbit Alexa-Fluor 488 (1:400; Jackson ImmunoResearch Labs). Note that for staining of LV-GFP-injected animals, GFP was visualized without staining. Images were acquired using a confocal microscope (LSM 510, Zeiss) at identical settings at the highest intensity for each of the conditions. c-Fos positive cells were counted and analyzed with Imaris 7.3.0 software (BITPLANE). The analysis was conducted on every 5th section in a series of 40 μm coronal sections throughout the dorsal DG, CA3 or CA1. Cells were considered positive for c-Fos-like immunoreactivity if the nucleus was the appropriate size and shape (at least 50% of circularity), and was distinct from the background.

**Analysis of ZL006 concentrations**

ZL006 (10 μM, 1 μl, 3.28 μg) was microinjected into the dorsal CA3, microdialysis of dorsal hippocampus was performed after 24 hours^9^. The mice were sacrificed by decapitation and the brain tissue was rapidly removed and blocked rapidly over ice into coronal sections. Serial hippocampal sections (400 μm) were made on a vibratome (Leica) in a bath of pre-cold PBS. Then the cannulas-implanted side was obtained in the first three pieces of hippocampal sections and placed in the pre-cooling saline. The brain tissue concentrations were determined using the HPLC method with ultraviolet detection. Chromatographic separation was performed using a reversed-phase (C_18_) stainless steel column. The mobile phase consisted of methanol-aqueous 30 mM HAc (54:45, v/v). The flow-rate was set at 1.0 ml min^-1^, and the sample size was fixed at 20 µl. The column temperature was maintained at 30℃. Wavelength was set at 284 nm. A concentrated stock solution of ZL006 (0.60 mg/ml) was prepared in methanol and was further diluted into 0.05–60 µg/ml with the supernatant from brain homogenate for the preparation of standard samples. All the solutions were stored at 4℃. For the analysis, the supernatant from brain homogenate (100 µl) and shaken on a vortex mixer for 3 min. After centrifuging at 10,000 rpm for 10 min, 20 µl of the supernatant liquid was injected into the HPLC system for analysis.

**Open-field test (OFT)**

The open field is a widely used test to observe the basic movement of mice^12-14^. Open-field activity assay was performed at 24 h after the NSF test using the Motor Monitor System SF16R (CA, USA) on PC computer. The test arena was constructed of a plastic plate (56.13 × 56.13 cm) and divided into 256 squares by lines drawn on the floor of the plate. It was surrounded by a 35.18 cm high plastic wall. Each mouse was placed onto a corner square of the arena, facing the corner and allowed to freely explore the open field for 5 min per trial. During the period, the numbers of entered outer and inner squares were counted. An entry into a square was defined as having the two forelimbs in the square at one time. After each trial the plate was cleaned with 70% EtOH.

1. Rezayof A., Shariﬁ K., Zarrindast MR. & Rassouli Y. Modulation of ethanol state-dependent learning by dorsal hippocampal NMDA receptors in mice. *Alcohol* **42**: 667-674 (2008).
2. AN XL & TAI FD. AVP and Glu systems interact to regulate levels of anxiety in BALB/ cJ mice. *Zoological Research* **35**: 319−325 (2014).
3. [de Carvalho Myskiw J](https://www.ncbi.nlm.nih.gov/pubmed/?term=de Carvalho Myskiw J[Author]&cauthor=true&cauthor_uid=24591622), Furini CR, Benetti F & Izquierdo I. Hippocampal molecular mechanisms involved in the enhancement of fear extinction caused by exposure to novelty. [*Proc Natl Acad Sci U S A*](https://www.ncbi.nlm.nih.gov/pubmed/24591622) **111**: 4572-4577 (2014).
4. Wu Q., Zheng RM , Srisai D. , McKnight GS. & Palmiter R. NR2B subunit of the NMDA glutamate receptor regulates appetite in the parabrachial nucleus. [*Proc Natl Acad Sci U S A*](https://www.ncbi.nlm.nih.gov/pubmed/24591622) **36**: 14765-14770 (2013).
5. Plotnikov1 A. *et al*. The nuclear translocation of ERK1/2 as an anticancer target. *Nat Commun* **10**: 1038 (2015).
6. Wang WS. *et al*. Extinction of aversive memories associated with morphine withdrawal requires ERK-mediated epigenetic regulation of brain-derived neurotrophic factor transcription in the rat ventromedial prefrontal cortex. *J Neurosci* **32**: 13763-13775 (2002).
7. Stepan J., Hladky F., Uribe A., Holsboer F., Schmidt MV. & Eder M. High-Speed imaging reveals opposing effects of chronic stress and antidepressants on neuronal activity propagation through the hippocampal trisynaptic circuit. *Front Neural Circuits* **9**: 70 (2015).
8. Luo CX. *et al*. Bidirectional regulation of neurogenesis by neuronal nitric oxide synthase derived from neurons and neural stem cells. *Stem Cells* **28**: 2041-2052 (2010).
9. Zhou L. *et al*. Treatment of cerebral ischemia by disrupting ischemia-induced interaction of nNOS with PSD-95. *Nat Med* **16**: 1439-1443 (2010).
10. Luo CX. *et al*. Interaction of nNOS with PSD-95 negatively controls regenerative repair after stroke. *J Neurosci* **34**: 13535-13548 (2014).
11. Powell KL. *et al*. Decreases in HCN mRNA expression in the hippocampus after kindling and status epilepticus in adult rats. *Epilepsia* **49**: 1686-1695 (2008).
12. Walsh RN & Cummins RA. The Open-Field Test: a critical review. *Psychol Bull* **83**:482-504 (1976).
13. Bruhwyler J, Chleide E, Liegeois JF, Delarge J & Mercier M. Anxiolytic potential of sulpiride, clozapine and derivatives in the open-field test. *Pharmacol Biochem Behav* **36**:57-61(1990).
14. Carola V, D'Olimpio F, Brunamonti E, Mangia F & Renzi P. Evaluation of the elevated plus-maze and open-field tests for the assessment of anxiety-related behaviour in inbred mice. *Behav Brain Res* **134**:49-57 (2002).

**Supplementary Figures and Legends**


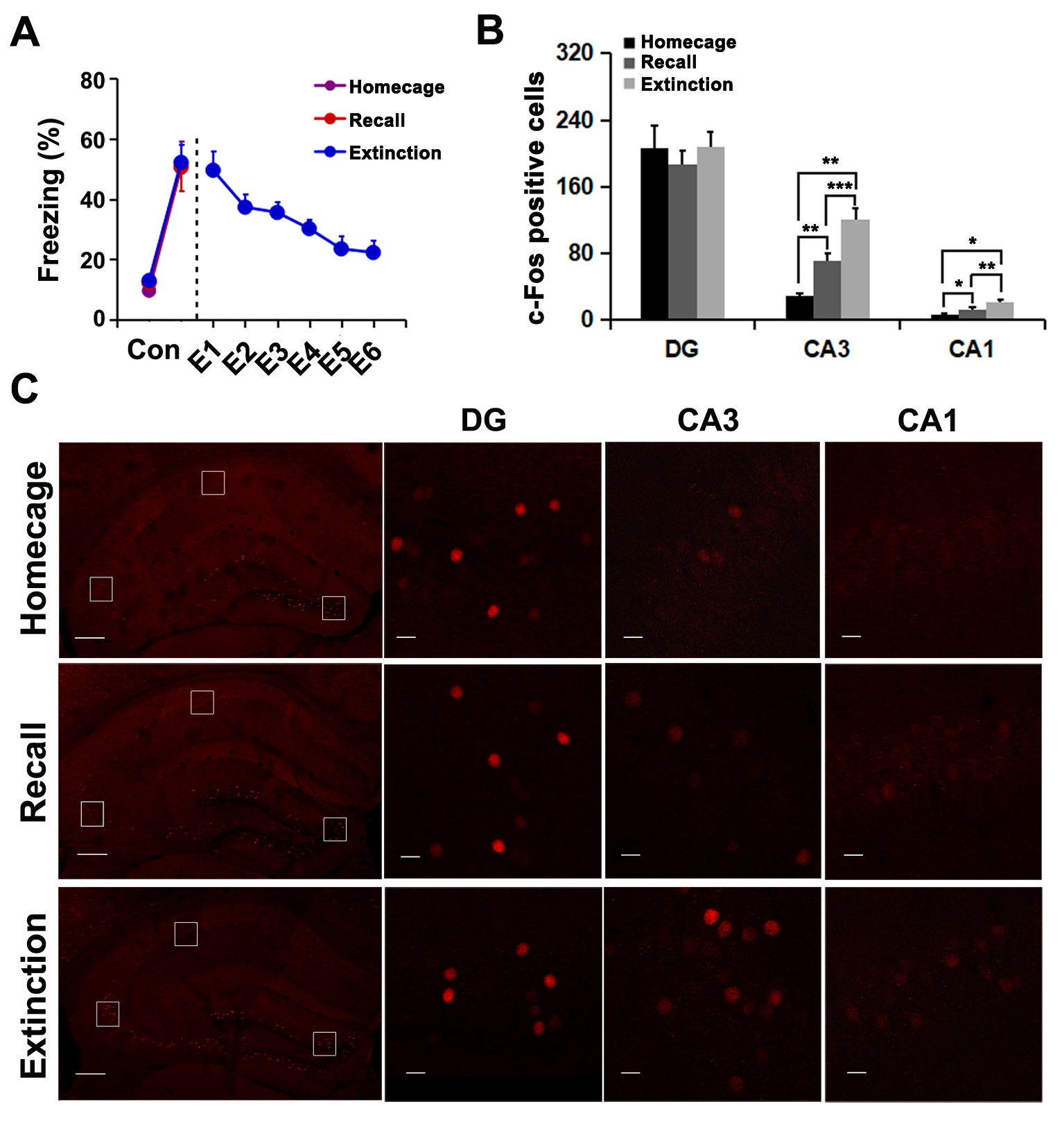


**Figure S1.** Contextual fear extinction triggers abundant c-Fos expression in the dorsal CA3. (**A**) Freezing behavior of mice measured during fear recall and extinction trial (n = 4-6-10). Fear conditioning consisted of a 3-min context exposure followed by a single electric foot shock (0.7 mA, constant current, 2 s). Fear recall was performed 24 h later by re-exposing the mice for 3 min into the conditioning context. Control groups did not subject to fear extinction remaining in their homecages (Homecage). The recall-group was perfused 90 min after contextual fear recall (Recall). Next day after recall, the mice were subjected to 6 extinction trials. Each extinction trial consisted of a 3 min re-exposure to the conditioned context without presenting the foot shock again. The extinction-group was perfused 90 min after contextual fear recall (Extinction). (**B**) There is no difference of the number of c-Fos-positive neurons among three groups (homecage *vs* recall, F(1,7) = 0.58, *p* = 0.4703; homecage *vs* extinction, F(1,7) = 0.00, *p* = 0.965; recall *vs* extinction, F(1,8) = 0.96, *p* = 0.355) in the DG. Contextual fear recall (F(1,7) = 22.46, *p* = 0.002) and contextual fear extinction (F(1,7) = 48.25, *p* < 0.001) increased the number of c-Fos-positive neurons in the CA3, and c-Fos-positive neurons numbers in extinction group were higher than the number in contextual fear recall group (F(1,8) = 13.42, *p* = 0.006). Contextual fear recall (F(1,7) = 6.02, *p* = 0.044) and contextual fear extinction (F(1,7) = 22.05, *p* = 0.002) increased the number of c-Fos-positive neurons in the CA1, and c-Fos-positive neurons numbers in extinction group were higher than the number in contextual fear recall group (F(1,8) = 7.43, *p* = 0.026). (**C**) Left: Representative fluorescence images showing c-Fos expression in the dorsal hippocampus of control groups not subjected to fear extinction remaining in their homecages (top), a group subjected to contextual fear recall (middle) and a group subjected to massed extinction (bottom). Scale bar = 200 μm. Right: The high-magnification images from a selected area in left. Scale bar = 5 μm.


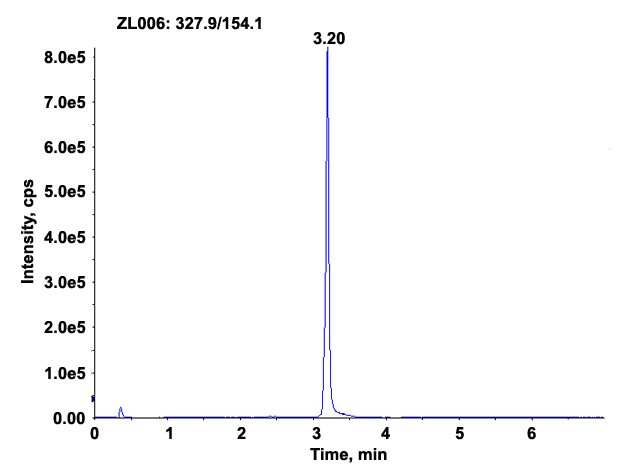


**Figure S2.** The [concentration](../../../../D:/%25E6%259C%2589%25E9%2581%2593/Dict/6.3.69.8341/resultui/frame/javascript:void(0)%3B) of ZL006 was detected in the dorsal hippocampus after 24 hours of intra-hippocampus infusions (10 μM, 1 μl, 3.28 μg) by LC-MS/MS analysis. (A) Represesentative traces showing chromatographic separation and detection of ZL006 (Concentration (ng/ml), n = 6, 135.70 ± 25.126).


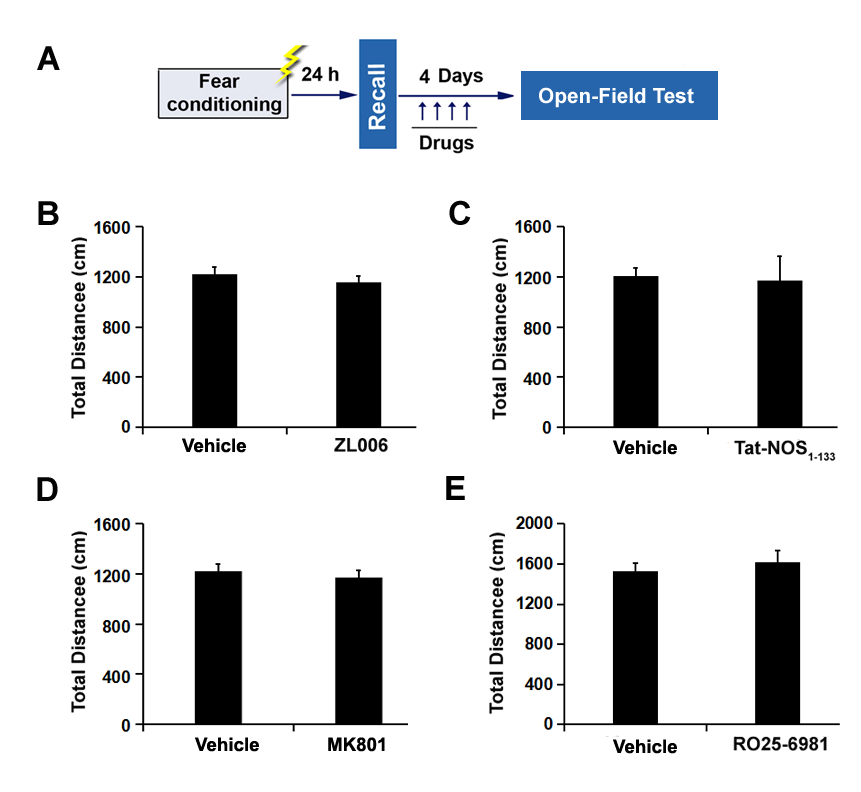


**Figure S3.** PSD-95-nNOS blockers and NMDARs antagonists did not change animal’s locomotion. **(A)** Design of the experiments for B, C, D, E. **(B)** Effect of intra-CA3 ZL006 for 4 days did not change animal’s locomotion in the Open-Field Test (n = 14, F(1,26) = 0.74, *p* = 0.3966). **(C)** Effect of intra-CA3 Tat-NOS_1-133_ for 4 days did not change animal’s locomotion in the Open-Field Test (n = 13, F(1,24) = 0.23, *p* = 0.6359). **(D)** Effect of intra-CA3 MK801 for 4 days did not change animal’s locomotion in the Open-Field Test. (n = 14-13, F(1,225) = 0.46, *p* = 0.5029). **(E)** Effect of intra-CA3 RO25-6981 for 4 days did not change animal’s locomotion in the Open-Field Test. (n = 12-11, F(1,21) = 0.45, *p* = 0.5093).


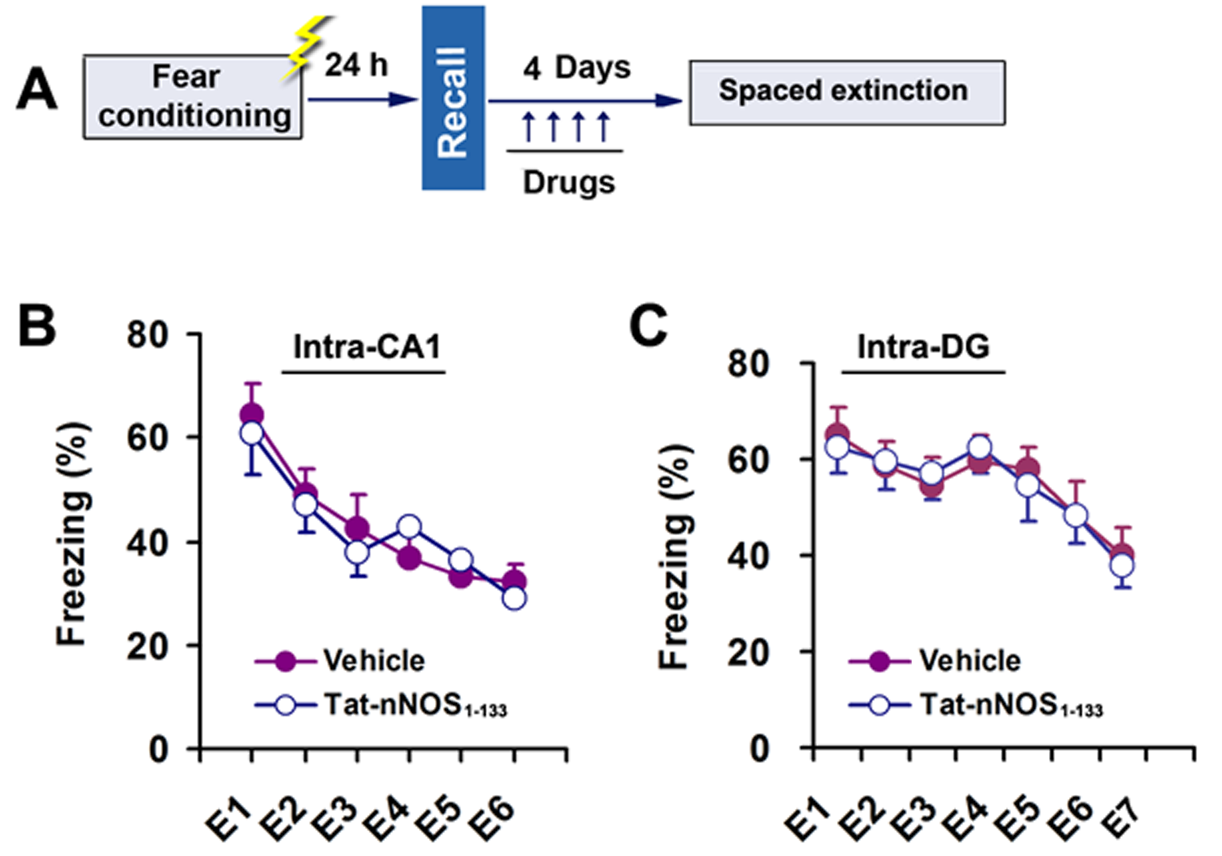


**Figure S4.** Disrupting PSD-95-nNOS interaction in the dorsal CA1 and DG has no effect on contextual fear extinction. (**A**) Design of the experiments for **B, C**. (**B**) Effect of intra-CA1 Tat-nNOS_1-133_ on contextual fear extinction. (**C**) Effect of intra-CA1 Tat-nNOS_1-133_ on contextual fear extinction.


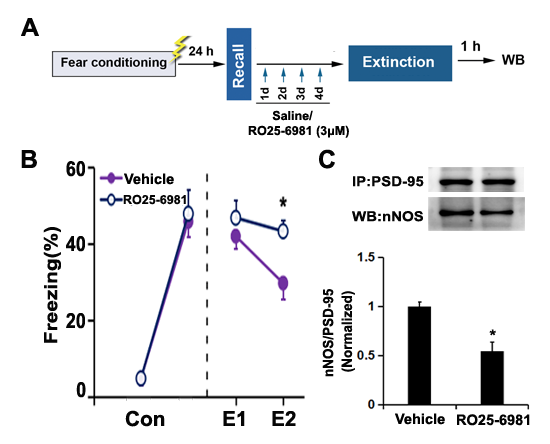


**Figure S5**. NMDARs antagonist down-regulates nNOS-PSD95 interaction. **(A)** Design of the experiments for B, C. **(B)** Freezing behavior measured during contextual fear conditioning and extinction trial (n = 9. E2, F(1,16) = 7.36, **p* = 0.015). **(C)** Immunoblots showing nNOS-PSD95 complex level in the dorsal CA3 treated by RO25-6981 (n = 6).


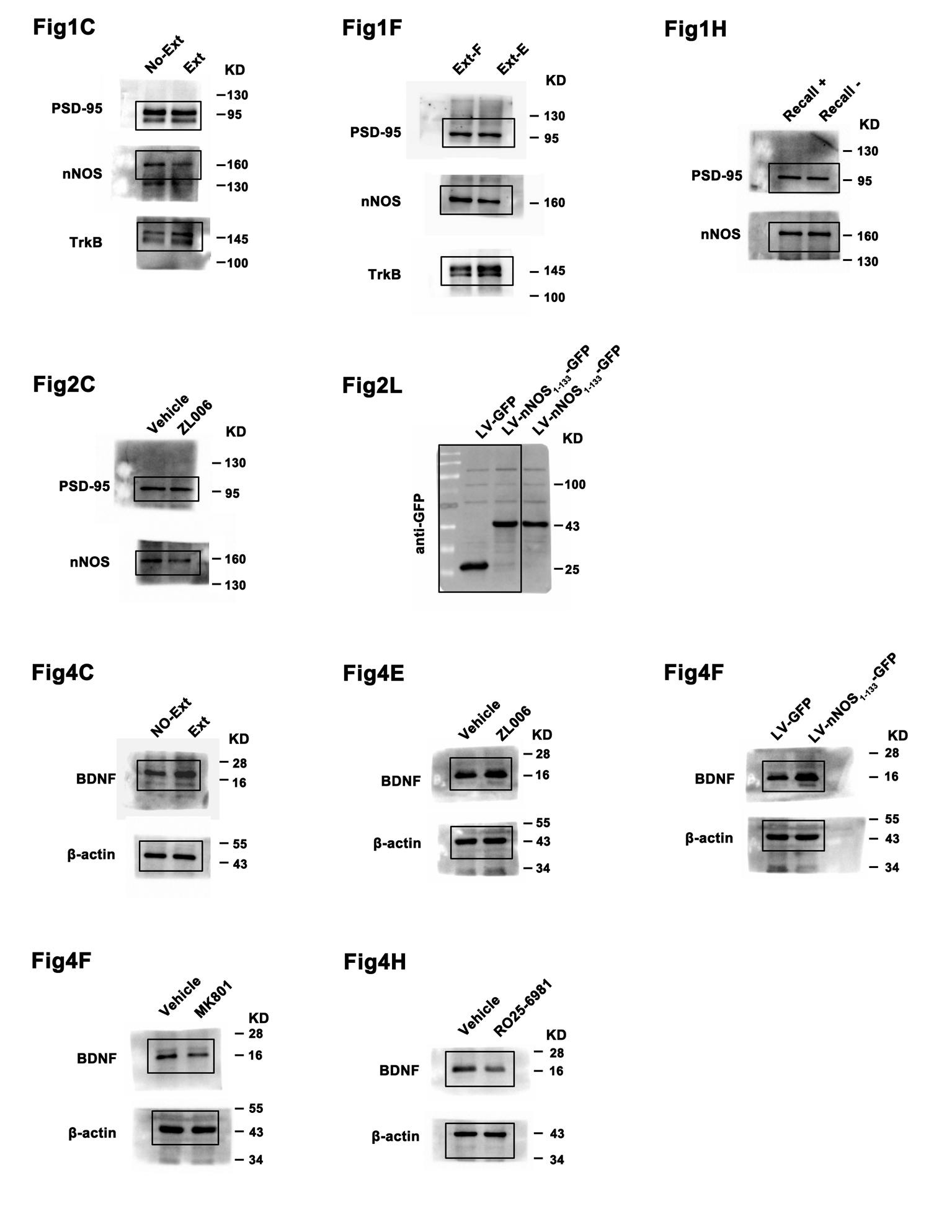


**Figure S6**. Full-length pictures of the blots presented in the main figures.


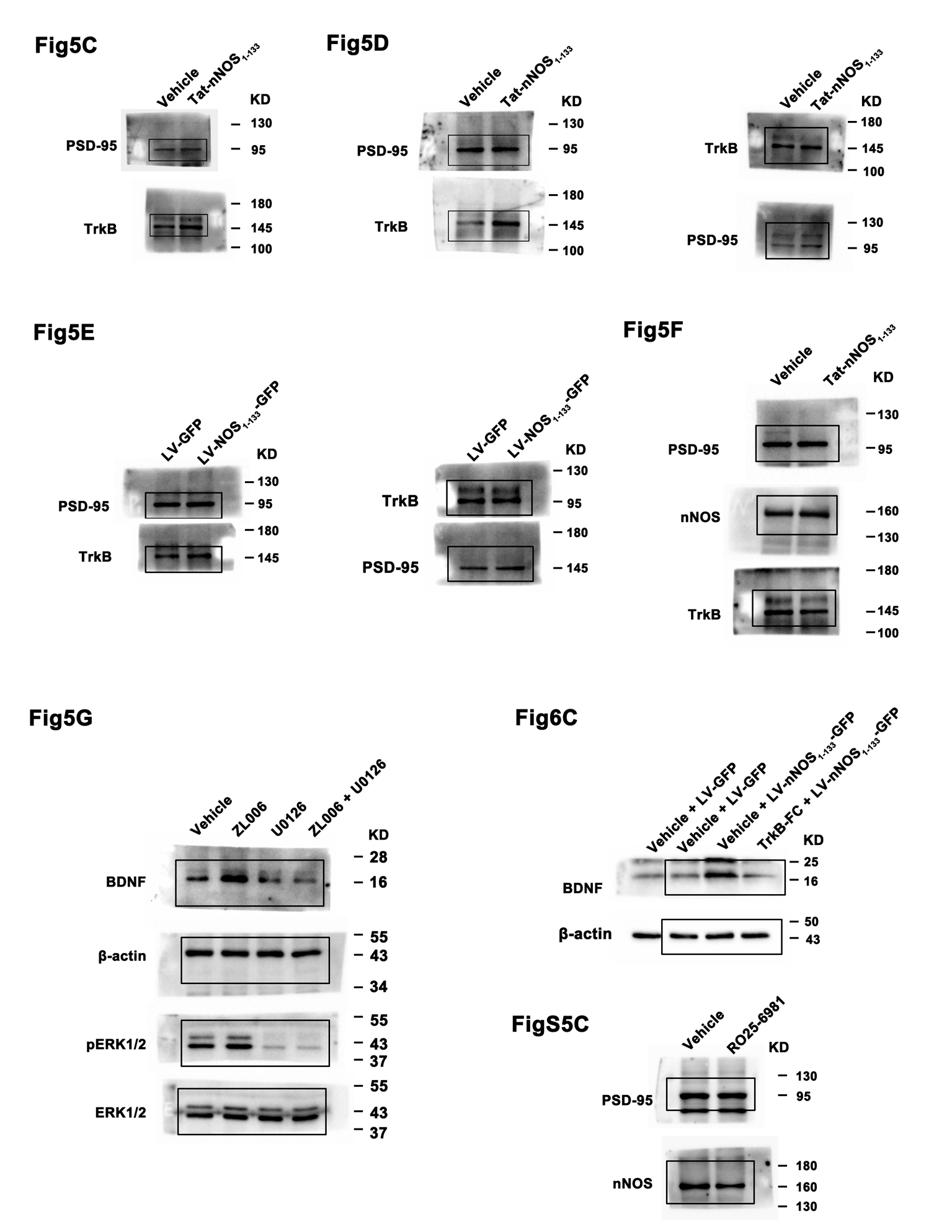


**Figure S7**. Full-length pictures of the blots presented in the main figures.

**Table S1**: The F- and P-values from repeated-measures analysis of variance (ANOVA) followed by Tukey’s *post hoc* test for behavioral experiments.

|  | Extinction | | Treatment | | Extinction × Treatment | |
| --- | --- | --- | --- | --- | --- | --- |
|  | F-values | P-values | F-values | P-values | F-values | P-values |
| *Fig1.B* | F(9,13) = 12.825 | *p* < 0.001 | - | - | - | - |
| *Fig1.E* | F(5,10) = 23.165 | *P* = 0.001 | - | - | - | - |
| *Fig2.B* | F(4,16) = 11.383 | *p* < 0.001 | F(1,16) = 4.96 | *p* = 0.019 | F(4,64) = 0.976 | *p* = 0.427 |
| *Fig2.F* | F(6,26) = 67.683 | *p* < 0.001 | F(6,26) = 4.96 | *p* = 0.376 | F(6,156) = 0.872 | *p* = 0.481 |
| *Fig2.G* | F(6,16) = 37.051 | *p* < 0.001 | F(1,16) = 11.171 | *p* = 0.004 | F(6,96) = 1.512 | *p* = 0.182 |
| *Fig2.H* | F(6,24) = 11.358 | *p* < 0.001 | F(1,24) = 20.177 | *p* < 0.001 | F(6,144) = 0.654 | *p* = 0.687 |
| *Fig2.I* | F(4,25) = 39.615 | *p* < 0.001 | F(1,25) = 5.268 | *p* = 0.030 | F(4,100) = 3.336 | *p* = 0.013 |
| *Fig2.M* | F(5,22) = 9.278 | *p* < 0.001 | F(5,22) = 4.444 | *p* = 0.047 | F(5,110) = 1.513 | *p* = 0.192 |
| *Fig2.N* | F(6,40) = 14.344 | *p* < 0.001 | F(5,22) = 7.004 | *p* = 0.001 | F(18,240) = 0.609 | *p* = 0.891 |
| *Fig3.B* | F(6,27) = 12.283 | *p* < 0.001 | F(6,27) = 4.889 | *p* = 0.036 | F(6,162) = 1.088 | *p* = 0.372 |
| *Fig3.C* | F(7,24) = 111.241 | *p* < 0.001 | F(7,24) = 4.286 | *p* = 0.049 | F(7,168) = 1.513 | *p* = 0.002 |
| *Fig3.D* | F(6,25) = 9.278 | *p* = 0.001 | F(6,25) = 8.229 | *p* = 0.008 | F(6,150) = 3.550 | *p* = 0.003 |
| *Fig4.B* | F(9,5) = 29.572 | *P* = 0.003 | - | *-* | - | *-* |
| *Fig4.F* | F(1,10) = 0.758 | *p* = 0.404 | F(1,10) = 5.369 | *p* = 0.043 | F(1,10) = 4.599 | *p* = 0.058 |
| *Fig4.G* | F(1,22) = 40.956 | *p* < 0.001 | F(1,22) = 4.728 | *p* = 0.041 | F(1,22) = 2.175 | *p* = 0.154 |
| *Fig4.H* | F(1,21) = 10.357 | *p* = 0.004 | F(1,21) = 4.606 | *p* = 0.044 | F(1,21) = 2.367 | *p* = 0.139 |
| *Fig6.B* | F(6,32) = 16.172 | *p* < 0.001 | F(2,32) = 5.933 | *p* = 0.006 | F(12,192) = 1.312 | *p* = 0.214 |
| *Fig6.D* | F(4,30) = 11.499 | *p* < 0.001 | F(2,30) = 8.580 | *p* = 0.004 | F(8,120) = 1.769 | *p* = 0.090 |
| *FigS4.B* | F(5,22) = 7.729 | *p* < 0.001 | F(5,22) = 0.009 | *p* = 0.923 | F(5,110) = 0.529 | *p* = 0.754 |
| *FigS4.C* | F(6,21) = 6.766 | *p* < 0.001 | F(6,21) = 0.002 | *p* = 0.969 | F(6,126) = 0.155 | *p* = 0.988 |
| *FigS5.B* | F(1,16) = 7.308 | *p* = 0.016 | F(1,16) = 6.791 | *p* = 0.019 | F(1,16) = 2.253 | *p* = 0.153 |
